# Antiplatelet therapy with aspirin, clopidogrel, and dipyridamole versus clopidogrel alone or aspirin and dipyridamole in patients with acute cerebral ischaemia (TARDIS): a randomised, open-label, phase 3 superiority trial

**DOI:** 10.1016/S0140-6736(17)32849-0

**Published:** 2018-03-03

**Authors:** Philip M Bath, Lisa J Woodhouse, Jason P Appleton, Maia Beridze, Hanne Christensen, Robert A Dineen, Lelia Duley, Timothy J England, Katie Flaherty, Diane Havard, Stan Heptinstall, Marilyn James, Kailash Krishnan, Hugh S Markus, Alan A Montgomery, Stuart J Pocock, Marc Randall, Annemarei Ranta, Thompson G Robinson, Polly Scutt, Graham S Venables, Nikola Sprigg, TARDIS Investigators, L M Christensen, L Bentsen, C Krarup Hansen, T T Thomsen, C Kruuse, H H Jensen, S S Hansen, V Petrovic, N Beridze, N Kakabadze, T Kherkheulidze, D Kakabadze, I Toidze, N Lobjanidze, N Akiashvili, A Tevdoradze, N Khizanishvili, T Tsanava, R Taylor, I Iniesta, J Kok, J Duignan, M Funnell, P Cariga, M Rodriguez, I J Watson, S Tennant, M Macleod, J Furnace, H Gow, J Irvine, A Joyson, S Nelson, V Taylor, M Smith, R Bellfield, B Hairsine, R Davies, A Dodd, J Corrigan, M Doherty, A Ahmed, C Denniss, S Johnson-Holland, K A Kay, R Icart Palau, G Auld, P Daboo, R Erande, G Grimwood, D Hove, L Howaniec, O Redjep, R Rangasamay, G Butt, D Sandler, J Reddan, S Stafford, J McIlmoyle, S Maguire, P Murphy, J Chambers, L Guthrie, M Osborn, A Steele, M Burn, A Benford, A Misra, D Hilton, E O'Brien, E Amis, S Finlay, J Mitchell, O Geraghty, K Harvey, B Hazel, S Mashate, P Wilding, M Sajid, M Ball, R Gascoyne, R Sivakumar, A Wright, K Chatterjee, S Booth, H Eccleson, C Kelly, S Leason, C Perkins, D Bruce, E Brown, S Clayton, M Garside, G Rogers, E Lawrence, S Mahmood, C Watchurst, D Chadha, L Glover, L Holford, K Smith, D Walstow, R Williams, L O'Shea, J Goodsell, C Athulathmudali, E Barbon, R Namushi, P Jacob, L Johnson, D Morse, C McGhee, O Speirs, S Atkinson, A Peacocke, P Langhorne, R Graham, F Wright, C McAlpine, A Ravindrane, M Bajoriene, L Matter, S Windebank, E Giallombardo, D Dellafera, C Eglinton, J Wilson, D Broughton, K Chapman, L Dixon, M Zaidi, K Ayes, J Kessell, D Manawadu, O Adegbaju, J Aeron-Thomas, K Anderson, A Brigden, E Cattermole, J Good, S Hassan, E Khoromana, L Lee-Carbon, K Marks, E Mckenzie, N Sikondari, M Cooper, K Whysall, I Wynter, J Bamford, A Hassan, P Wanklyn, M Kambafwile, L Makawa, D Waugh, E Veraque, MGG Soliman, S Arif, R Brown, S Butler, C Hewitt, J Hindle, A Pusalkar, H Beadle, K Chan, M Siddiqui, P Dangri, S Buddha, A Asokanathan, A Mistri, D Eveson, K Musarrat, L Manning, S Anand, P Christian, S Khan, C Patel, M Sein, J Banns, E Gibson, T Gordon, Y Gruenbeck, S Wong, P Datta, G Bateman, L Jackson, A Needle, Y Duodu, R Oliver, C Padilla-Harris, M Barber, D Esson, F Brodie, C McInnes, K Fotherby, D Butler, D Morgan, K Preece, A Willberry, M Dent, F Hammonds, J Hunt, C Vernon, D O'Kane, F Faola, P Lai, J O'Callaghan, C Smith, C Price, R Lakey, V Riddell, A Smith, G Storey, S Munshi, A Buck, J Clarke, N Gilzeane, M Godfrey, R Keshvara, C Richardson, J Roffe, L Ryan, F Shelton, W Sunman, A Tittle, J Tomlinson, K Whittamore, G Wilkes, P Owusu-Agyei, N Temple, D Mangion, A Hardwick, K Netherton, A Mohd Nor, B Hyams, S Norman, N Persad, S Ragab, C Dickson, J Dube, E Jinks, K Knops, B Wadams, K Ali, J Gaylard, G Spurling, L Sztriha, T Ajao, M Alao, F K Chan, P Webster, P Howard, T J Dobson, L Hyatt, D Sims, J Cunningham, B Esisi, T Cassidy, M Bokhari, B McClelland, B Mokoena, G Gunathilagan, S Jones, M Reader, G Thomas, S Tilby, P Findlay, F Barrett, F Leslie, S Ross, I Shread, J Okwera, J Howe, F Harrington, G Courtauld, C Schofield, R Donnelly, M Maddula, J Scott, J Beavan, K Muhidden, I Memon, M Clarke, A Hedstrom, L Mills, A Hemsley, A Bowring, L Boxall, H Kingwell, S Keenan, C Roughan, A Manoj, P Cox, G Fletcher, P Lopez, H Emsley, B Gregary, A McLoughlin, S Raj, C Roffe, N Abano, A Barry, A Butler, R Carpio, K Castro, K Finney, S Gomm, J Hiden, J Grocott, S Lyjko, H Maguire, A Remegoso, R Sanyal, S Stevens, I Natarajan, J Chembala, G Muddegowda, A Warusevitane, A Blight, O Balazikova, C Lawlor, L Shaw, D Button, D Howcroft, S Lucas, B Madigan, S McCann, A Dixit, A Barkat, J Davis, M Fawcett, L Finlay, H Guy, C Hays, V Hogg, E Horsley, C Hubbuck, C Pringle, C Stevenson, K Storey, T Thompson, S Woodward, A Banerjee, C Allcock, S Merotra, C Douglass, E Campbell, R Jarapa, M Johnes, C Keaveney, T Marsden, Z Naing, J Perez, K Shaw, T Black, A Anthony, C Clarke, J Paterson, K Deighton, E Temlett, C Blank, C Doyle, S Duty, K Gill, K Harkness, C Kamara, E Richards, K Elfandi, P Guyler, P Harman, C Khuoge, S Kunhunny, S Tysoe, B Moynihan, T Adedoyin, N Chopra, N Dayal, R Ghatala, N Jeyaraj, I Jones, F Kennedy, L Kerin, N Khanom, S Lewis, S Maheswaran, L Montague, M Niemierko, J O'Reilly, S Trippier, F Watson, P Wilkinson, E Young, K Dizayee, H Cochrane, J O'Connell, L Mokoena, E Osborne, A Nair, J Greig, C Jenkins, J Powell, F Price, M Chowdhury, S Brixey, L Hunt, N Rands, G Rose, S Stoddart, M Srinivasan, N Motherwell, R Shekhar, T Fuller, A Lankester, P Lingwood, C Rankin, H Webb, B Jupp, J Bell, G Hann, B Longland, C Ovington, B Bhaskaran, G Ayres, C Bailey, H Bearne, J Buxton, P Fitzell, C Hilaire, D Kelly, S Szabo, D Tomlin, E Gamble, B Charles, R Kumar, T Fluskey, Z Mellor, J Peters, V Sutton, A Kenton, I Martin, S Nyabadza, S Ghosh, M Henry, B Kumar, D Bruce, C Ambulo, S Crawford, T Nozedar, M Platton, V Cvoro, M Couser, K McCormick, D Wilkinson, K Javaid, S Hurdowar, T Attygalle, S Sundayi, O Orugun, H Crowther, R Jolly, U Poultney, A Azim, M Krasinska-Chavez, J White, N Sengupta, J Margalef, M G Metiu, S Meenakshisundaram, S Dealing, D Hargroves, E Beranova, L Cowie, H Rudenko, A Thomson, A Verrion, K Rashed, S Board, C Buckley, D Hayward, K Jenkins, E Keeling, R Rowland-Axe, C Vickers, D Wood, A Lehman, R Erande, M Patel, H Russell, H Rehman, D Forrest, P Farren

**Affiliations:** a Stroke Trials Unit, Division of Clinical Neuroscience, University of Nottingham, Nottingham, UK; b Radiological Sciences, Division of Clinical Neuroscience, University of Nottingham, Nottingham, UK; c Nottingham Clinical Trials Unit, University of Nottingham, Nottingham, UK; d Vascular Medicine, Division of Medical Sciences & GEM, University of Nottingham, Nottingham, UK; e Health Economics, Division of Rehabilitation and Ageing, University of Nottingham, Nottingham, UK; f Stroke, Nottingham University Hospitals NHS Trust, City Hospital Campus, Nottingham, UK; g Hospital of War Veterans, Tbilisi, Georgia; h Bispebjerg and Frederiksberg Hospital, University of Copenhagen, Department of Neurology, Copenhagen, Denmark; i Stroke Research Group, Department of Clinical Neurosciences, University of Cambridge, Cambridge Biomedical Campus, Cambridge, UK; j Department of Medical Statistics, London School of Hygiene & Tropical Medicine, London, UK; k Department of Neurology, Leeds General Infirmary, Leeds Teaching Hospitals NHS Trust, Leeds, UK; l Department of Neurology, Wellington Hospital and University of Otago, Wellington, New Zealand; m Department of Cardiovascular Sciences and NIHR Leicester Cardiovascular Research Centre, University of Leicester, Leicester, UK; n Department of Neurology, Sheffield Teaching Hospitals NHS Foundation Trust, Royal Hallamshire Hospital, Sheffield, UK

## Abstract

**Background:**

Intensive antiplatelet therapy with three agents might be more effective than guideline treatment for preventing recurrent events in patients with acute cerebral ischaemia. We aimed to compare the safety and efficacy of intensive antiplatelet therapy (combined aspirin, clopidogrel, and dipyridamole) with that of guideline-based antiplatelet therapy.

**Methods:**

We did an international, prospective, randomised, open-label, blinded-endpoint trial in adult participants with ischaemic stroke or transient ischaemic attack (TIA) within 48 h of onset. Participants were assigned in a 1:1 ratio using computer randomisation to receive loading doses and then 30 days of intensive antiplatelet therapy (combined aspirin 75 mg, clopidogrel 75 mg, and dipyridamole 200 mg twice daily) or guideline-based therapy (comprising either clopidogrel alone or combined aspirin and dipyridamole). Randomisation was stratified by country and index event, and minimised with prognostic baseline factors, medication use, time to randomisation, stroke-related factors, and thrombolysis. The ordinal primary outcome was the combined incidence and severity of any recurrent stroke (ischaemic or haemorrhagic; assessed using the modified Rankin Scale) or TIA within 90 days, as assessed by central telephone follow-up with masking to treatment assignment, and analysed by intention to treat. This trial is registered with the ISRCTN registry, number ISRCTN47823388.

**Findings:**

3096 participants (1556 in the intensive antiplatelet therapy group, 1540 in the guideline antiplatelet therapy group) were recruited from 106 hospitals in four countries between April 7, 2009, and March 18, 2016. The trial was stopped early on the recommendation of the data monitoring committee. The incidence and severity of recurrent stroke or TIA did not differ between intensive and guideline therapy (93 [6%] participants *vs* 105 [7%]; adjusted common odds ratio [cOR] 0·90, 95% CI 0·67–1·20, p=0·47). By contrast, intensive antiplatelet therapy was associated with more, and more severe, bleeding (adjusted cOR 2·54, 95% CI 2·05–3·16, p<0·0001).

**Interpretation:**

Among patients with recent cerebral ischaemia, intensive antiplatelet therapy did not reduce the incidence and severity of recurrent stroke or TIA, but did significantly increase the risk of major bleeding. Triple antiplatelet therapy should not be used in routine clinical practice.

**Funding:**

National Institutes of Health Research Health Technology Assessment Programme, British Heart Foundation.

## Introduction

The risk of recurrence after ischaemic stroke and transient ischaemic attack (TIA) is highest immediately after the event and declines over the following weeks.^1^ Although aspirin reduces the risk of early recurrence,^2^, ^3^ dual antiplatelet therapy might be more effective, with the choice of antiplatelets less important than the use of two agents rather than one (at least when considering drugs with different modes of action such as aspirin, clopidogrel, and dipyridamole).^4^ Subsequently, the Clopidogrel in High-Risk Patients with Acute Nondisabling Cerebrovascular Events (CHANCE) trial^5^, ^6^ found that combined aspirin and clopidogrel was superior to aspirin alone in preventing recurrence by 90 days in Chinese patients with minor ischaemic stroke or TIA when randomly assigned within 24 h of onset.

If dual therapy is superior to monotherapy for acute secondary prophylaxis, then intensive short-term treatment with three antiplatelet agents might be better still, providing the absolute risk of recurrence is high, there is a beneficial reduction in recurrence with treatment, and the risk of bleeding on treatment does not become excessive. In proof-of-mechanism and proof-of-concept studies, triple therapy was more effective than single or dual agents in inhibiting platelet aggregation, platelet–leucocyte conjugation, and leucocyte activation in vitro^7^ and ex vivo in healthy volunteers and participants with previous stroke or TIA.^8^, ^9^ A small trial in participants with chronic stroke reported that combined aspirin, clopidogrel, and dipyridamole (compared with aspirin alone) was feasible to administer for up to 24 months, although bleeding was increased with intensive treatment.^10^ In a case series, long-term administration of triple treatment appeared to be useful in participants at very high risk of recurrence, defined as recurrence on dual antiplatelet therapy.^11^

**Research in context**

**Evidence before this study**

We searched PubMed for relevant articles in November, 2012, with search terms “antiplatelet therapy”, “aspirin”, “dipyridamole”, “clopidogrel”, “ticlopidine”, “prasugrel”, “cilostazol”, “triflusal”, “glycoprotein IIb/IIIa receptor antagonists”, “stroke”, “cerebral ischemia”, “cerebral infarction”, “transient ischemic attack”, and “randomized controlled trial”. We also manually searched references from original articles and pertinent reviews. Searches were restricted to completed trials in human beings with abstracts or full texts published. The risk of recurrence after ischaemic stroke and transient ischaemic attack (TIA) is highest immediately after the event, and is reduced with aspirin. Acute dual antiplatelet therapy appears to be more effective according to the CHANCE trial. A meta-analysis that included CHANCE found that dual antiplatelet therapy significantly reduced the risk of stroke recurrence (risk ratio 0·69, 95% CI 0·60–0·80; p<0·001) and the composite outcome of stroke, TIA, acute coronary syndrome, and all death (0·71, 0·63–0·81; p<0·001) when compared with monotherapy, and non-significantly increased the risk of major bleeding (1·35, 0·70–2·59, p=0·37). If two agents are superior to one, then we hypothesised that intensive treatment with three might be better, if the absolute risk of recurrence is high, there is a beneficial reduction in recurrence with treatment, and the risk of bleeding on treatment does not become excessive.

**Added value of this study**

TARDIS is the only large trial of intensive antiplatelets testing the combination of three agents. Intensive antiplatelet therapy with three drugs did not reduce the incidence and severity of recurrent stroke or TIA, but it did significantly increase the risk of major bleeding when compared with guideline antiplatelet therapy. Composite endpoints of any stroke, fatal haemorrhage, or major haemorrhage, and death, stroke, myocardial infarction, fatal haemorrhage, or major haemorrhage did not differ between the treatment groups.

**Implications of all the available evidence**

Triple antiplatelet therapy should not be used in routine clinical practice for secondary prevention after ischaemic stroke or TIA.

The Triple Antiplatelets for Reducing Dependency after Ischaemic Stroke (TARDIS) trial compared the safety and efficacy of intensive versus guideline-based antiplatelet therapy in patients with acute non-cardioembolic ischaemic stroke or TIA.

## Methods

### Study design and participants

TARDIS was an international, multicentre, prospective, randomised, open-label, blinded-endpoint, superiority trial done in 106 sites in four countries (Denmark, Georgia, New Zealand, and the UK). Details of the study rationale and design, statistical analysis plan, and characteristics of the participants have been described elsewhere.^12^, ^13^, ^14^ The protocol is available online.

Adult patients presenting to hospital in four countries were eligible for inclusion if they were at risk of a recurrent ischaemic stroke and had either a non-cardioembolic ischaemic stroke with limb weakness, dysphasia, or neuroimaging-positive hemianopia, or a non-cardioembolic TIA with at least 10 min of limb weakness or isolated dysphasia. Participants had to be randomly assigned within 48 h of symptom onset. Participants who received intravenous thrombolysis could be randomly assigned but only after 24 h had elapsed after the end of this treatment, and providing post-treatment neuroimaging excluded secondary cerebral bleeding.

Key exclusion criteria were age younger than 50 years; isolated sensory symptoms, facial weakness, or vertigo or dizziness; presumed cardioembolic stroke or TIA; parenchymal haemorrhage or other intracranial haemorrhage; non-ischaemic cause for symptoms; definite need for, or contraindication to, aspirin, clopidogrel, or dipyridamole; definite need for full-dose anticoagulation; premorbid dependency; or severe hypertension. A full list of the study inclusion and exclusion criteria is provided in the appendix.

Patients gave written consent, or written proxy consent was obtained from a relative or carer if the patient lacked capacity. The study was approved by national or local ethics committees in each participating country and site and was adopted in the UK by the National Institute of Health Research Stroke Research Network. National competent authorities (or equivalent) gave approvals for this study. The trial was overseen by a trial steering committee (including five independent members and a patient-public representative) and an international advisory committee (comprising each national coordinator). The day-to-day conduct of the trial was run by a trial management committee based at the Stroke Trials Unit in Nottingham. An independent data monitoring committee reviewed unblinded data in confidence every 6 months.

### Randomisation and masking

Demographic and baseline clinical characteristics were entered online into a secure web-based database system that also provided randomisation. Baseline data were checked to confirm the patient's eligibility and the system then assigned the participant to intensive or guideline antiplatelet therapy, with allocation in a 1:1 ratio (appendix p 9).

Treatment assignment comprised stratification by country and index event (stroke *vs* TIA) and minimisation^15^ on key prognostic baseline factors (age, sex, premorbid function, systolic blood pressure, syndrome [cortical *vs* lacunar and posterior^16^], previous antiplatelet therapy [none or monotherapy *vs* dual therapy], use of gastroprotection, use of low-dose heparin, and time to randomisation). Minimisation also included presence of crescendo TIAs (more than one TIA in previous week) and ABCD2 score^17^ for participants with TIA, and National Institutes of Health Stroke Scale (NIHSS) and treatment with alteplase for those with stroke. Minimisation included a random element in 5% of patients.

Final follow-up was done centrally at 90 days by telephone from the coordinating centre in each country, with the assessor masked to treatment allocation. If the participant could not be contacted, a questionnaire covering the same outcome measures was sent by post.

### Procedures

Participants randomly assigned to the intervention group received combined aspirin (300 mg load then 50–150 mg daily, typically 75 mg, given by oral, nasogastric, or rectal routes), clopidogrel (300 mg load then 75 mg daily, given by oral or nasogastric routes), and dipyridamole (200 mg twice daily modified release, given orally, or 100 mg three or four times daily, given by oral or nasogastric route). Those randomly assigned to guideline antiplatelet therapy received either combined aspirin and dipyridamole, or clopidogrel alone (more details are provided in the appendix pp 9–10), using the same loading and maintenance doses as in the intervention group. Randomly assigned antiplatelet drugs were given for 30 days after which participants were treated according to local guidelines, typically with clopidogrel alone or combined aspirin and dipyridamole. Drugs were sourced by each participating hospital and could involve any manufacturer (including generic sources).

### Outcomes

The primary efficacy outcome was the incidence and severity of recurrent stroke and TIA during follow-up to 90 days. Severity was assessed using a six-level ordinal scale:^13^, ^18^ fatal stroke, non-fatal severe stroke (modified Rankin Scale [mRS] 4 or 5), moderate stroke (mRS 2 or 3), mild stroke (mRS 0 or 1), TIA, and neither stroke nor TIA.^12^, ^13^ The mRS^19^ is a measure of dependency and scores range from zero to six, with a score of zero indicating no symptoms, five indicating severe dependency, and six indicating death.

Prespecified secondary outcomes at day 90 included activities of daily living (Barthel Index [BI]), cognition (modified telephone Mini-Mental State Examination [t-MMSE], Telephone Interview for Cognition Scale-modified [TICS-M], and categorical verbal fluency using animal naming), health-related quality of life (European Quality of Life-5 dimensions-3 level [EQ-5D-3L], from which health status utility value [HSUV] was calculated, and EQ-Visual Analogue Scale [EQ-VAS]), and mood (short Zung Depression Score [ZDS]). At discharge from initial hospital admission, duration of hospital stay and discharge destination (to institution or home) were recorded.

The main safety outcome was haemorrhage on a five-level ordinal scale: fatal, major, moderate, minor, and none.^18^ We defined fatal, major, and moderate haemorrhage according to the International Society on Thrombosis and Haemostasis, based on severity, site of bleeding, fall in haemoglobin, and need for transfusion.^20^ Additional safety outcomes included all-cause and cause-specific case fatality, early neurological deterioration (defined as an increase from baseline to day 7 of at least four points on the NIHSS, or decrease in consciousness in the NIHSS consciousness domain), and serious adverse events.

To assess the net balance between efficacy and hazard, we analysed composite endpoints of any stroke or major haemorrhage (including fatal haemorrhage); and death, stroke, myocardial infarction, or major haemorrhage.

Participants were seen in clinic at days 7 (on treatment) and 35 (end of treatment plus 5–7 days to allow for washout) to ascertain whether any outcome or bleeding events had taken place (including a full blood count) and to determine compliance with treatment. Identification of recurrent cerebrovascular events was triangulated between investigator reporting at days 7 and 35 and through serious adverse events, patient reporting at day 90 telephone follow-up, and by the general practitioner via a questionnaire posted to them shortly after day 90. The primary outcome, haemorrhage, and investigator-reported serious adverse events (including cause-specific case-fatality) were validated and categorised by expert adjudicators who were masked to treatment assignment. Participants who did not receive their assigned treatment or who did not adhere to the protocol were still followed up in full at day 90 and included in all analyses.

### Statistical analyses

The null hypothesis was that intensive antiplatelet therapy would not alter recurrence or severity of recurrence in participants with acute ischaemic stroke or TIA. The alternative hypothesis was that stroke and TIA recurrence and severity would differ between participants randomly assigned to intensive antiplatelet therapy versus those assigned to guideline antiplatelet therapy. Using an ordinal analysis, we estimated the overall sample size at 4100 participants to detect a shift in the distribution of the primary outcome with a common odds ratio [cOR] of 0·68 (representing the odds of a patient on treatment moving to a more severe category of outcome compared with a patient on control), two-sided type I error of 5%, power of 90%, dropout frequency of 2%, treatment crossover of 5%, and adjustment for baseline covariates.^21^ The sample calculation was not affected materially by planned interim analyses by the data monitoring committee after recruitment and follow-up of 40% and 70% of patients; the efficacy stopping rule was set at p less than 0·001 for the combined outcome of fatal stroke, non-fatal stroke, or major bleeding.

We analysed the effect of treatment on the primary efficacy outcome as a shift in stroke and its severity, with adjustment for the factors used in stratification and minimisation at the time of randomisation,^15^ reported as an adjusted cOR with 95% CIs. The odds ratio and significance were calculated with ordinal logistic regression following a check (using the likelihood ratio test) that the assumption of common proportional odds was not violated. For sensitivity purposes, the primary outcome was also analysed without adjustment, and as a binary outcome of fatal or major stroke versus neither. The heterogeneity of the treatment effect on the primary outcome was assessed in prespecified subgroups by adding an interaction term in an unadjusted ordinal logistic regression model. Similarly, the effect of treatment on the main safety outcome was analysed as a shift in bleeding and its severity (fatal, major, moderate, minor, or none) with adjustment for the stratification and minimisation factors. For sensitivity purposes, ordinal bleeding was analysed unadjusted and as a binary outcome of fatal and major haemorrhage; heterogeneity was also assessed. The composite outcomes of stroke or major haemorrhage—and death, stroke, myocardial infarction, or major haemorrhage—were compared between treatment groups with adjusted Cox regression.

We analysed death with Kaplan-Meier and Cox regression models. Other outcomes were analysed with adjusted multiple linear regression (BI, ZDS, t-MMSE, TICS-M, verbal fluency, EQ-5D3L-HSUV, and EQ-VAS). Because outcomes such as mRS, EQ-5D3L-HSUV, and BI include death (scores of 6, 0, and −5, respectively), and in case treatment was associated with asymmetric effects on death and other outcome measures (eg, more death but less impairment), we added an extreme value for death to the other outcome scales (−1 for EQ-VAS, −1 for t-MMSE, 43 for NIHSS, −1 for TICS-M, −1 for verbal fluency, and 102·5 for ZDS).^22^, ^23^

All analyses were done adjusted and unadjusted for completeness. The nominal level of significance for all analyses, including interaction testing, was p less than 0·05. No adjustment was made for multiplicity of testing for secondary analyses. Data are shown as number (%), median (IQR), mean (SD) and either mean difference, hazard ratio (HR), or cOR with 95% CIs. All analyses were by the intention-to-treat principle for all comparisons, including safety analyses. A per-protocol analysis was also done on the primary outcome. Statistical analyses were done according to the published statistical analysis plan^13^ by LJW and KF (with oversight by SJP) using SAS software version 9.3.

### Role of the funding source

The trial was conceived and designed by the grant applicants who wrote the protocol. Study data were collected, monitored, and analysed in Nottingham at the Stroke Trials Unit. Analysis, interpretation, and report writing were done independently of the funders and sponsor; no pharmaceutical companies were involved in any part of the trial. The corresponding author and two statisticians (LJW, KF) had full access to all the data in the study; additionally, the corresponding author had final responsibility for the decision to submit for publication and is the guarantor for the study. The funders had no role in study design, data collection, data analysis, data interpretation, or writing of the report. The writing committee also approved the decision to submit the report for publication.

## Results

**Figure 1:**
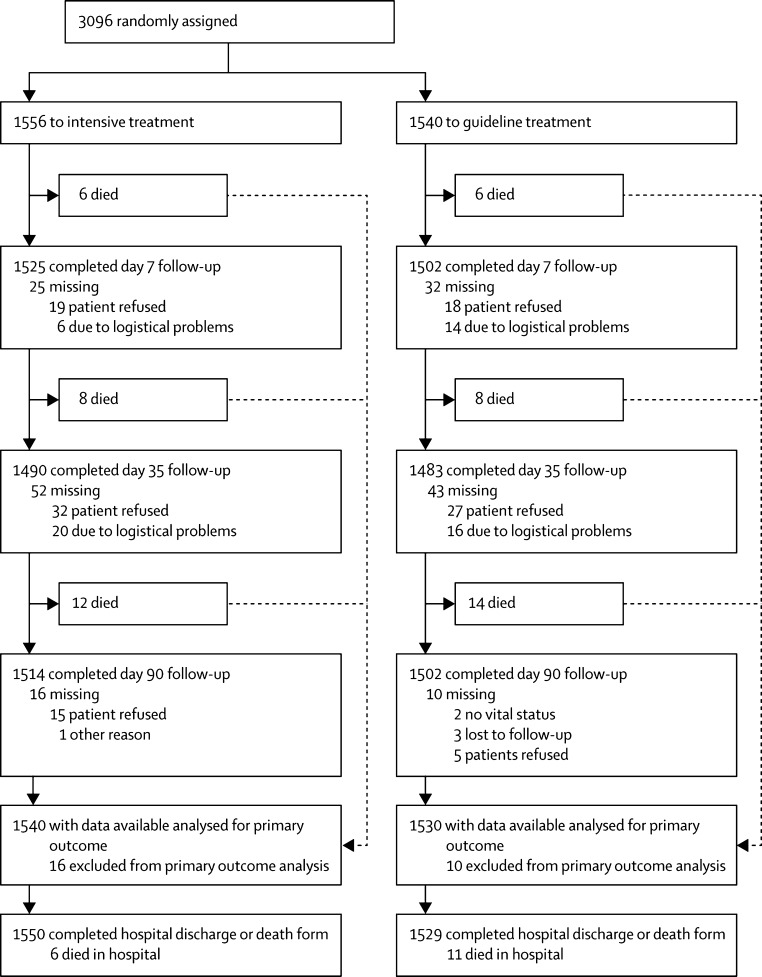
Study flow Screening for eligibility was not collected routinely.

Recruitment commenced on April 7, 2009, and on the advice of the independent data monitoring committee was halted on March 18, 2016, after enrolment of 3096 participants (76% of the planned target of 4100; figure 1). 28 patients in the intensive treatment group and 30 in the guideline treatment group were classified as inpatients in hospital—participants who were already admitted to hospital when they had their qualifying event. Most participants were recruited in the UK (2955 [95%]; table 1). The mean age was 69·0 years (SD 10·1) and 1945 (63%) participants were male. Investigations after randomisation judged the qualifying event to be ischaemic stroke in 2220 (72%) patients, TIA in 838 (27%) patients, and neither ischaemic stroke nor TIA in 38 (1%). The median time from onset to randomisation was 29·3 h (IQR 21·8–39·6); 314 (10%) participants were recruited within 12 h of onset and 651 (21%) were recruited within 13–24 h. In those with an ischaemic stroke, the mean NIHSS was 4·0 (SD 3·8) and 336 (16%) of 2143 patients who were initially randomised received thrombolysis. In those with TIA, median ABCD2 score was 5·0 (IQR 5·0–6·0) and 155 (20%) of 953 patients initially randomised presented with a crescendo TIA (defined as more than one TIA over the previous week). Among all participants, mean blood pressure was 143·5 mm Hg (SD 18·2) systolic and 79·5 mm Hg (11·4) diastolic, and the clinical syndrome was cortical in 1593 (51%) patients, lacunar in 1288 (42%), and posterior in 213 (7%).

**Table 1:** Baseline characteristics

			**Total (n=3096)**	**Intensive antiplatelet therapy (n=1556)**	**Guideline antiplatelet therapy (n=1540)**
Age, years*			69·0 (10·1)	69·1 (9·9)	68·9 (10·3)
Sex*					
	Male		1945 (63%)	982 (63%)	963 (63%)
	Female		1151 (37%)	574 (37%)	577 (37%)
Geographical region†					
	UK		2955 (95%)	1482 (95%)	1473 (96%)
	Denmark		51 (2%)	26 (2%)	25 (2%)
	Georgia		83 (3%)	45 (3%)	38 (2%)
	New Zealand		7 (<1%)	3 (<1%)	4 (<1%)
Medical history					
	Previous antiplatelet agents				
		Aspirin	816 (26%)	412 (26%)	404 (26%)
		Aspirin and dipyridamole	85 (3%)	43 (3%)	42 (3%)
		Clopidogrel	162 (5%)	89 (6%)	73 (5%)
		Other	17 (1%)	13 (1%)	4 (<1%)
	Previous heparin		7 (<1%)	2 (<1%)	5 (<1%)
	Hypertension†		1824 (59%)	930 (60%)	894 (58%)
	Hyperlipidaemia		1317/2973 (44%)	655/1496 (44%)	662/1477 (45%)
	Atrial fibrillation‡		1 (<1%)	0	1 (<1%)
	Stroke		348 (11%)	189 (12%)	159 (10%)
	Ischaemic heart disease		403 (13%)	196 (13%)	207 (13%)
	Peripheral artery disease		70 (2%)	40 (3%)	30 (2%)
Current smoker			784 (26%)	404 (26%)	380 (25%)
Qualifying event†					
	Ischaemic stroke		2220 (72%)	1121 (72%)	1099 (71%)
	TIA		838 (27%)	413 (27%)	425 (28%)
		Crescendo§¶	155/773 (20%)	72/388 (19%)	83/385 (22%)
		Patients on dual antiplatelet therapy before having their TIA¶	36 (4%)	23 (6%)	13 (3%)
	Non-ischaemic stroke or TIA||		38 (1%)	22 (1%)	16 (1%)
Weakness			2789 (90%)	1392 (89%)	1397 (91%)
Sensory loss			1066 (34%)	511 (33%)	555 (36%)
Dysphasia			1007 (33%)	522 (34%)	485 (31%)
	Isolated		160 (5%)	88 (6%)	72 (5%)
Neglect			331 (11%)	154 (10%)	177 (11%)
Hemianopia			304 (10%)	146 (9%)	158 (10%)
	Isolated		16 (1%)	6 (<1%)	10 (1%)
NIHSS (out of 42)*			2·8 (3·6)	2·9 (3·7)	2·7 (3·5)
ABCD2 score (out of 7)*			5·0 (5·0–6·0)	5·0 (5·0–6·0)	5·0 (5·0–6·0)
OCSP classification*					
	Number of patients		3094	1556	1538
	Total anterior		181 (6%)	86 (6%)	95 (6%)
	Partial anterior		1412 (46%)	714 (46%)	698 (45%)
	Lacunar		1288 (42%)	646 (42%)	642 (42%)
	Posterior		213 (7%)	110 (7%)	103 (7%)
TOAST**					
	Cardioembolic‡		134 (4%)	65 (4%)	69 (4%)
	Large vessel		490 (16%)	268 (17%)	222 (15%)
	Small vessel		1224 (40%)	621 (40%)	603 (40%)
	Mixed		22 (1%)	8 (1%)	14 (1%)
	Other/undetermined		1182 (39%)	569 (37%)	613 (40%)
Blood pressure					
	Systolic, mm Hg*		143·5 (18·2)	143·4 (17·8)	143·6 (18·5)
	Diastolic, mm Hg		79·5 (11·4)	79·4 (11·3)	79·6 (11·5)
Brain imaging					
	Number of patients		3092	1555	1537
	Normal or no lesion		1550 (50%)	770 (50%)	780 (51%)
	Ischaemic stroke		1390 (45%)	702 (45%)	688 (45%)
	Non-stroke lesion		6 (<1%)	4 (<1%)	2 (<1%)
	No brain scan		146 (5%)	79 (5%)	67 (4%)
Time from onset to randomisation, h*			29·3 (21·8–39·6)	29·3 (21·7–39·7	29·3 (21·9–39·5)
	Ischaemic stroke		32·1 (24·7–41·2)	32·2 (24·6–41·7)	32·0 (24·8–41·0)
	TIA		24·2 (17·5–29·7)	24·3 (17·5–29·5)	24·2 (17·5–30·0)
Time from onset to randomisation					
	≤12 h		314 (10%)	147 (9%)	167 (11%)
	13–24 h		651 (21%)	342 (22%)	309 (20%)
	>24 h		2131 (69%)	1067 (69%)	1064 (69%)
Thrombolysis*			341 (11%)	169 (11%)	172 (11%)

Data are number (%), median (IQR), or mean (SD). TIA=transient ischaemic attack. NIHSS=National Institutes of Health Stroke Scale. OCSP=Oxfordshire Community Stroke Project. TOAST=Trial of ORG 10 172 in Acute Stroke Treatment.

* Minimisation variable.

† Stratification variable.

‡ Protocol violation.

§ More than one TIA in previous week.

¶ Participants with TIA only.

|| One patient was enrolled with a TIA without previous scan, deteriorated after treatment, and on scanning was found to have an intracerebral haemorrhage.

** Participants with ischaemia only (n=3052); 38 patients had their qualifying event reclassified as a non-ischaemic event, resulting in a total of 3058 patients with ischaemia. Six of the 3058 patients had missing TOAST data, resulting in 3052 patients with ischaemia only.

The advice by the data monitoring committee to prematurely terminate recruitment was based on a combination of three observations: intensive antiplatelet therapy was associated with a significant increase in major (including fatal) bleeding; intensive antiplatelet therapy was not associated with a significant reduction in the primary outcome; and a conditional power analysis suggested the trial was highly unlikely to demonstrate a significant difference in the primary outcome were it to continue. The trial steering committee reviewed the same data as well as supplementary analyses on April 12, 2016, and noted additionally that there was no difference in the net balance of death, stroke, myocardial infarction, and major bleeding. They agreed that the trial should stop recruitment on the basis of futility.

**Figure 2:**
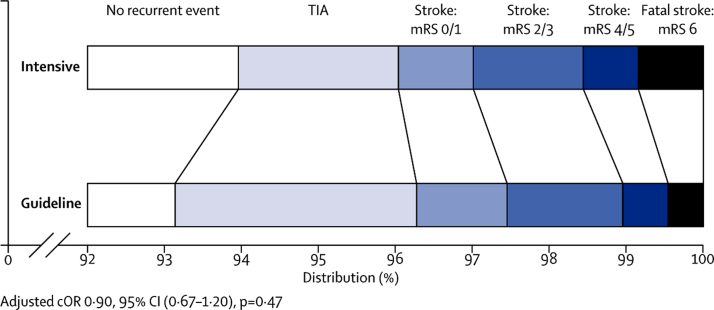
Distribution of recurrent stroke and TIA by severity The primary outcome was incidence and severity of stroke (fatal, mRS 4–5, mRS 2–3, mRS 0–1) and TIA at day 90. TIA=transient ischaemic attack. mRS=modified Rankin Scale.

Final follow-up (at day 90) was completed for 3016 (97%) participants and vital status was available for all but five (<1%; figure 1). Outcomes were determined by telephone in 2889 (93%) participants and by post in 67 (2%). The primary outcome (further stroke or TIA and function at day 90) was determined in 3070 (99%) participants. Overall, 198 (6%) participants had a recurrent stroke or TIA (93 in the intensive therapy group *vs* 105 in the guideline therapy group; table 2), comprising 118 strokes (96 ischaemic, 19 haemorrhagic, five of unknown type due to absence of neuroimaging, one patient with both ischaemic and haemorrhagic stroke, and one patient with an ischaemic and an unknown type of stroke) and 80 TIAs (of the 88 patients who had a TIA, eight also had a recurrent stroke; when deriving the primary outcome, the most severe event was counted). There was no difference in the incidence and severity of stroke or TIA (using the primary efficacy outcome of ordered categorical scale comprising fatal stroke, stroke with mRS 4 or 5, stroke with mRS 2 or 3, stroke with mRS 0 or 1, or TIA) between intensive and guideline antiplatelet therapies (adjusted cOR 0·90, 95% CI 0·67–1·20, p=0·47; table 2, figure 2). When the primary outcome was assessed in prespecified subgroups (appendix p 26), no significant interactions between the primary outcome and treatment were present; in the intensive arm there was a tendency for fewer and less severe strokes or TIAs in participants presenting with a mild stroke and worse outcomes in participants with more severe stroke, but this interaction was not significant (p=0·070). In a sensitivity analysis, the frequency of stroke or TIA by day 90 did not differ between participants receiving intensive versus guideline antiplatelet therapy (adjusted HR 0·87, 95% CI 0·66–1·16, p=0·34; table 2, appendix p 27). However, patients receiving intensive treatment were less likely to have a TIA than those on guideline therapy (adjusted HR 0·63, 95% CI 0·41–0·97, p=0·034).

**Table 2:** Efficacy outcomes

		**Intensive antiplatelet therapy (n=1556)**	**Guideline antiplatelet therapy (n=1540)**	**Adjusted cOR or HR (95% CI)**	**p value**
**Primary outcome**					
Number of patients		1540	1530	··	··
Ordinal stroke or TIA		93 (6%)	105 (7%)	0·90 (0·67–1·20)	0·47
	Death (mRS 6)	13 (1%)	7 (<1%)	1·92 (0·76–4·84)	0·17
	mRS 4–5	11 (1%)	9 (1%)	··	··
	mRS 2–3	22 (1%)	23 (2%)	··	··
	mRS 0–1	15 (1%)	18 (1%)	··	··
	TIA	32 (2%)	48 (3%)	··	··
	No stroke or TIA	1447 (94%)	1425 (93%)	··	··
**Sensitivity analyses**					
Ordinal, per protocol		65/1089 (6%)	59/1007 (6%)	1·07 (0·74–1·55)	0·72
Stroke or TIA		93/1540 (6%)	105/1530 (7%)	0·87 (0·66–1·16)	0·34
Stroke		61/1540 (4%)	57/1530 (4%)	1·05 (0·73–1·51)	0·79
	Ischaemic	46/1540 (3%)	50/1530 (3%)	0·89 (0·59–1·33)	0·56
	Haemorrhagic	14/1540 (1%)	5/1530 (<1%)	2·77 (0·99–7·75)	0·052
	Unknown	2/1540 (<1%)	3/1530 (<1%)	0·47 (0·05–4·50)	0·51
	mRS >2	34/1540 (2%)	28/1530 (2%)	1·19 (0·72–1·97)	0·50
TIA		34/1540 (2%)	54/1530 (4%)	0·63 (0·41–0·97)	0·034
Death		26/1556 (2%)	28/1535 (2%)	0·89 (0·51–1·55)	0·69

Data are number (%), cOR (95% CI) for ordinal stroke or TIA, and for ordinal, per protocol analysis; HR (95% CI) for all other analyses. Comparisons by binary logistic regression, ordinal logistic regression, or Cox proportional hazards models with adjustment for baseline factors. Stroke or TIA is given by severity; when a patient had more than one event over 90 days, the most severe event is used. mRS=modified Rankin Scale. cOR=common odds ratio. HR=hazard ratio. TIA=transient ischaemic attack.

There was no difference between the treatment groups in outcome at day 90 assessed as disability (BI), mood (ZDS), cognition (t-MMSE, TICS-M, verbal fluency), or quality of life (EQ-5D3L-HSUV, EQ-VAS; appendix p 18). Analyses based on unadjusted comparisons did not differ qualitatively for either the primary or any of the secondary outcomes (data not shown).

The distribution of risk and severity of haemorrhage (using the ordinal scale of fatal, major, moderate, mild, or no haemorrhage) was shifted to more bleeding and bleeding of greater severity in participants randomly assigned to intensive antiplatelet therapy (adjusted cOR 2·54, 95% CI 2·05–3·16, p<0·0001; table 3, appendix p 28). When the distribution of haemorrhage and its severity was assessed in prespecified subgroups, a statistically significant interaction between haemorrhage and the type of comparator was present; intensive treatment was associated with more bleeding when compared with aspirin and dipyridamole than when compared with clopidogrel. An interaction was also seen for patients who received thrombolysis; intensive antiplatelet therapy was associated with more bleeding in those who received thrombolysis than those who did not (appendix p 29).

**Table 3:** Safety outcomes by treatment group

		**Intensive antiplatelet therapy (n=1556)**	**Guideline antiplatelet therapy (n=1540)**	**Adjusted cOR or HR (95% CI)**	**p value**
**Bleeding (safety analysis)**					
Ordinal bleeding (cOR)		305/1541 (20%)	139/1531 (9%)	2·54 (2·05–3·16)	<0·0001
	Fatal^20^	8/1541 (1%)	3/1531 (<1%)	3·48 (0·89–13·63)	0·074
	Major	31/1541 (2%)	14/1531 (1%)	··	··
	Moderate	25/1541 (2%)	13/1531 (1%)	··	··
	Mild	241/1541 (16%)	109/1531 (7%)	··	··
	None	1236/1541 (80%)	1392/1531 (91%)	··	··
**Sensitivity analyses**					
Fatal or major^20^		39/1540 (3%)	17/1530 (1%)	2·23 (1·25–3·96)	0·0063
Intracranial bleeding		16/1540 (1%)	5/1530 (<1%)	3·14 (1·14–8·61)	0·026
	Intracerebral	13/1540 (1%)	4/1530 (<1%)	3·26 (1·05–10·06)	0·040
	Subdural or extradural	2/1540 (<1%)	0	··	NC
	Fatal	6/1540 (<1%)	3/1530 (<1%)	2·43 (0·59–10·01)	0·22
	Major	9/1540 (1%)	1/1530 (<1%)	8·79 (1·10–69·95)	0·040
	Fatal or major	15/1540 (1%)	4/1530 (<1%)	3·84 (1·26–11·63)	0·018
Extracranial bleeding		293/1541 (19%)	135/1531 (9%)	2·37 (1·93–2·91)	<0·0001
	Gastrointestinal	48/1540 (3%)	34/1530 (2%)	1·39 (0·89–2·16)	0·15
	Other	255/1541 (17%)	104/1531 (7%)	2·70 (2·14–3·39)	<0·0001
	Fatal	2/1540 (<1%)	0	··	NC
	Major	24/1540 (2%)	13/1530 (1%)	1·71 (0·86–3·38)	0·13
	Fatal or major	26/1540 (2%)	13/1530 (1%)	1·89 (0·96–3·71)	0·064
Stroke or major bleeding		87/1540 (6%)	69/1530 (5%)	1·24 (0·90–1·70)	0·19
Death, stroke, myocardial infarction, or major bleeding		102/1540 (7%)	98/1530 (6%)	1·02 (0·77–1·35)	0·88
Serious adverse events* (cOR)		335/1543 (22%)	327/1531 (21%)	1·02 (0·86–1·22)	0·80
	Fatal	13/1543 (1%)	22/1531 (1%)	0·52 (0·25–1·05)	0·070
	Severe	54/1543 (4%)	39/1531 (3%)	··	··
	Moderate	167/1543 (11%)	148/1531 (10%)	··	··
	Mild	101/1543 (7%)	118/1531 (8%)	··	··
	None	1208/1543 (78%)	1204/1531 (79%)	··	··

Data are number (%), cOR (95% CI) for those analyses indicated and HR (95% CI) for the remaining analyses. The population of patients in this table was determined by the number of patients with recorded events plus the number of patients without events who completed final follow-up. Comparisons by Cox proportional hazards models or ordinal logistic regression with adjustment for baseline factors. Haemorrhage is most severe, not first, bleed over 90 days. No subarachnoid haemorrhages occurred. cOR=common odds ratio. HR=hazard ratio. NC=not calculable.

* Information on serious adverse events was available for 3074 participants.

17 (1%) participants receiving guideline antiplatelet therapy had severe (fatal or major) haemorrhage by day 90, compared with 39 (3%) receiving intensive antiplatelet therapy (adjusted HR 2·23, 95% CI 1·25–3·96, p=0·0063; table 3). The rates of bleeding increasingly diverged between the treatment groups up to the end of treatment at day 30, but not thereafter (appendix pp 19, 30). Combined fatal and major intracranial bleeding was increased with intensive antiplatelet therapy (adjusted HR 3·84, 95% CI 1·26–11·63, p=0·018). A non-significant tendency to more fatal or major extracranial bleeding was also seen with intensive antiplatelet therapy (adjusted HR 1·89, 95% CI 0·96–3·71, p=0·064; table 3).

Vital status at end of trial was available for 3091 participants. There was no evidence of a mortality difference between the treatment groups (table 2, appendix p 31). Excluding primary outcome and bleeding events, the overall occurrence of serious adverse events was similar in the two treatment groups (335 [22%] in the intensive group *vs* 327 [21%] in the guideline group; adjusted cOR 1·02, 95% CI 0·86–1·22, p=0·80; table 3, appendix pp 20, 32); similarly, the occurrence of fatal serious adverse events did not differ between the treatment groups (13 [1%] in the intensive group *vs* 22 [1%] in the guideline group; adjusted HR 0·52, 95% CI 0·25–1·05, p=0·070).

The composite endpoint of any stroke, fatal haemorrhage, or major haemorrhage occurred in 87 (6%) participants in the intensive group and 69 (5%) participants in the guideline group (adjusted HR 1·24, 95% CI 0·90–1·70, p=0·19; table 3). Similarly, the composite endpoint of death, stroke, myocardial infarction, fatal haemorrhage, or major haemorrhage did not differ between the treatment groups (102 [7%] in the intensive group *vs* 98 [6%] in the guideline group; adjusted HR 1·02, 95% CI 0·77–1·35, p=0·88).

## Discussion

In this cohort of patients with acute, non-cardioembolic ischaemic stroke or TIA, a regimen of intensive antiplatelet therapy did not reduce stroke recurrence or its severity when compared with guideline antiplatelet therapy with either clopidogrel alone or combined aspirin and dipyridamole. However, intensive antiplatelet therapy was associated with both more, and more severe, bleeding. There was no difference in mortality or the composite endpoint of stroke or major haemorrhage.

Previous meta-analyses of trials of antiplatelets in acute stroke and TIA have suggested that it is the number of drugs (ie, two *vs* one), rather than which ones, that is important when determining efficacy, at least when considering aspirin, clopidogrel, and dipyridamole.^4^, ^6^ If two antiplatelet agents are better than one, then three might be better still, providing that bleeding is not overly increased. However, TARDIS demonstrated that treatment with three agents does not reduce recurrent stroke but does increase haemorrhage. Because the primary outcome included haemorrhagic stroke, the failure to reduce stroke recurrence and its severity overall seems to reflect the combination of increased secondary intracranial haemorrhage and a tendency to reduced cerebral ischaemic events. Several factors appear to explain the results. First, participants with a severe stroke (typically cortical strokes) tended to do better on guideline therapy whereas intensive antiplatelet therapy favoured those with mild stroke. Although the risk of recurrence might be greater in mild stroke than in severe stroke, the explanation in TARDIS is not obvious because stroke severity did not seem to influence the effect of treatment on bleeding. Second, the type of guideline comparator appeared to be important since there was a tendency, albeit non-significant, for intensive therapy to have beneficial effects on the primary outcome in comparison with combined aspirin and dipyridamole, but not when compared with clopidogrel alone. In parallel, intensive antiplatelet therapy was more likely to cause bleeding when compared with combined aspirin and dipyridamole than when compared with clopidogrel. Nevertheless, these comparisons are indirect because most sites did not elect to randomly assign participants between the guideline groups.

A confounding factor was the use of thrombolysis, which might have increased the difference in bleeding between the intensive and guideline antiplatelet therapy groups. Trials such as CHANCE and the Acute Stroke or Transient Ischaemic Attack Treated with Aspirin or Ticagrelor and Patient Outcomes (SOCRATES) study excluded patients who received thrombolysis due to concerns about haemorrhage, particularly relevant since recruitment had to be within 24 h of ictus.^5^, ^24^ In TARDIS, randomly assigned antiplatelet therapy was commenced 24 h after the completion of any alteplase maintenance dose that might have promoted bleeding. A treatment–thrombolysis interaction was present for bleeding, which is surprising because the circulating half-life of alteplase is a few minutes (although the tissue and biological half-lives might be longer). Further, antiplatelet agents were given with a loading dose following thrombolysis, as recommended in guidelines, and it is possible that this acceleration of antiplatelet activity contributed to the risk of bleeding in the presence of recent thrombolysis. Nonetheless, this finding suggests that bleeding risk is higher following intravenous thrombolysis despite an interval of 24 h or more between completing alteplase and randomisation into the TARDIS study.

To our knowledge, TARDIS is the first trial designed to use ordered categorical primary and safety outcomes according to fatal event, severe non-fatal event, mild non-fatal event, or no event. Empirical analyses of this approach with published data from existing trials of antiplatelet therapies and other prophylactic interventions suggested that it would have more statistical power, or the same power for a smaller sample size.^18^ A key secondary aim of TARDIS was to test this methodological approach and to compare it with analyses based on binary outcomes. Because the primary efficacy analysis was non-significant, the relative merits of ordinal versus binary analysis could not be adequately assessed for stroke. However, ordinal analysis of bleeding gave similar, if not more pronounced, results in comparison with the outcome of fatal or major bleeding.

The present trial has several strengths, especially generalisability due to its wide inclusion criteria. In addition to motor presentations, patients with acute ischaemic stroke included those with severe stroke, dysphasia, or neuroimaging-positive hemianopia. Similarly, patients with TIA included those with crescendo TIA or who were already on dual antiplatelet agents. Hence, groups of patients that are typically excluded in stroke prevention trials could be enrolled. Inclusion of patients with severe stroke meant that those with cortical syndromes, often a minority in such trials, could participate. The wide time window of 48 h meant that patients could be enrolled after intravenous thrombolysis. Furthermore, the trial had a large sample size of more than 3000 patients, concealment of treatment assignment, prospective assessment of multiple outcomes including safety measures such as haemorrhage, very high follow-up (99% of participants had their primary outcome determined), care in specialist stroke services, and use of locally sourced aspirin, clopidogrel, and dipyridamole from a variety of manufacturers (thus increasing the external validity of the trial). An additional strength is that the results define clearly that although one or two agents are safe and effective in acute cerebral ischaemia, three agents do not add further efficacy.

Nevertheless, several limitations apply. First, the broad population might have included groups more likely to either respond (eg, those with minor stroke or TIA, or atherosclerotic disease^25^) or have a major bleed (eg, those receiving thrombolysis, or having small vessel disease^26^), which might explain the neutral results; future trials of antiplatelets might need to be more specific and focus on individuals with atherosclerotic disease. Second, the antiplatelet agents were administered in an open-label design and participants knew which drugs they were on. This aspect could have driven the reporting of known adverse events such as headache with dipyridamole and bleeding with intensive antiplatelet therapy. In mitigation, outcomes at day 90 were assessed centrally and masked to treatment assignment to reduce the potential for bias. Third, the comparator group involved different antiplatelet agents, a situation reflecting changes in national and international guidelines that added monotherapy with clopidogrel to the existing recommendation of combined aspirin and dipyridamole. The PRoFESS mega-trial^27^ compared these two strategies in 20 332 patients with chronic (not acute) ischaemic stroke or TIA and showed no differential effect on stroke recurrence, although major haemorrhage occurred more frequently with combined aspirin and dipyridamole. It should be noted that TARDIS did not allow aspirin monotherapy (as shown to be effective for ischaemic stroke in two mega-trials^2^, ^3^) as a comparator because it was not recommended in UK guidelines for secondary prevention in either 2005 or 2010, largely because both aspirin with dipyridamole, and clopidogrel alone, had previously been shown to be superior to aspirin alone in three large trials.^28^, ^29^, ^30^ Fourth, randomly assigned treatments were given for 30 days, which might have been too long in view of the identified haemorrhage risk. Importantly, the risk of haemorrhage for intensive and guideline antiplatelet therapy diverged from the start of randomised treatment and was significantly different by 14 days; it is not apparent that a shorter period of intensive antiplatelets would have avoided the risk of bleeding (appendix pp 13, 19). Last, the trial was stopped early following recommendation by the independent data monitoring committee and the results could represent a false neutral finding related to the lower-than-planned statistical power. However, the trial recruited more than 70% of its planned target of 4100 participants and the post-hoc statistical power remained high at 85%. Further, the prespecified effect size of 0·68 was almost ruled out (with 95% confidence). As such, it is likely that the trial's main findings are correct—ie, that intensive antiplatelet therapy does not appear to reduce recurrent cerebral ischaemic events but does increase the risk of haemorrhage.

In conclusion, findings from TARDIS show that among patients with acute ischaemic stroke or TIA who were recruited within 48 h after symptom onset, treatment with intensive antiplatelet therapy as compared with guideline antiplatelet therapy did not reduce stroke recurrence or its severity but did increase haemorrhage and its severity. Hence, intensive antiplatelet therapy based on the use of three routinely available drugs cannot be recommended in acute cerebral ischaemia.

For the **web-based database system** see https://nottingham.ac.uk/∼nszwww/tardis/tardistrialdb/tardis_login.php
