# Supplementary material for: Antiplatelet therapy with aspirin, clopidogrel, and dipyridamole versus clopidogrel alone or aspirin and dipyridamole in patients with acute cerebral ischaemia (TARDIS): a randomised, open-label, phase 3 superiority trial
Source: Lancet. 2018 Mar 3;391(10123):850–9. doi: 10.1016/S0140-6736(17)32849-0 (PMC5854459; doi:10.1016/S0140-6736(17)32849-0)

THE LANCET

Supplementary appendix

This appendix formed part of the original submission and has been peer reviewed. We post it as supplied by the authors.

Supplement to: Bath PM, Woodhouse LJ, Appleton JP, et al. Antiplatelet therapy with aspirin, clopidogrel, and dipyridamole versus clopidogrel alone or aspirin and dipyridamole in patients with acute cerebral ischaemia (TARDIS): a randomised, open-label, phase 3 superiority trial. *Lancet* 2017; published online Dec 20. http://dx.doi.org/10.1016/S0140-6736(17)32849-0.

SUPPLEMENTARY APPENDIX

TABLE OF CONTENTS

	Page
Title	1
Study Investigators	1
Methods	6
List of Tables and Figures	11
Tables	12
Figures	15
References	24

Title

Intensive versus guideline antiplatelet therapy in patients with acute cerebral ischaemia (TARDIS): a randomised, open-label, phase III, superiority trial

* Study Investigators

Writing Committee

Philip M Bath, Lisa J Woodhouse, Jason P Appleton, Maia Beridze, Hanne Christensen, Robert A Dineen, Lelia Duley, Timothy J England, Katie Flaherty, Diane Havard, Stan Heptinstall, Marilyn James, Kailash Krishnan, Hugh S Markus, Alan A Montgomery, Stuart Pocock, Marc Randall, Annemarei Ranta, Thompson Robinson, Polly Scutt, Graham S Venables, Nikola Sprigg; on behalf of the TARDIS Trialists

Trial Steering Committee

Independent members: Helen Rodgers (Newcastle, TSC Chair), Ahamad Hassan (Leeds), Christine Roffe (Stoke-on-Trent), Craig Smith (Salford), William Toff (Leicester)

Grant holders: Philip Bath (Nottingham, Chief Investigator), Rob Dineen (Nottingham, Neuroradiology Lead), Lelia Duley (Nottingham), Stan Heptinstall (Nottingham, Platelet Expert), Marilyn James (Nottingham, Health Economic Lead), Hugh Markus (Cambridge), Stuart Pocock (London, Statistical Lead), Thompson Robinson (Leicester), Nikola Sprigg (Nottingham, Deputy Chief Investigator), Graham Venables (Sheffield)

Patient-public representative (Nottingham): Oswald Newell (2008-14), Chibeka Kasonde (2014-16)

Sponsor's representative: Angela Shone (University of Nottingham)

International Advisory Committee

Denmark - Hanne Christensen (Copenhagen), Georgia - Maia Beridze (Tblisi), New Zealand - Anna Ranta (Wellington), UK – Philip Bath (Chair, Nottingham)

Data Monitoring Committee

Ian Ford (Glasgow, UK; Chair), Didier Leys (Lille, France), Cathie Sudlow (Edinburgh, UK), Matthew Walters (Glasgow,

UK)

Independent Events (outcome, SAE) Adjudicator

Nikola Sprigg (Nottingham, UK), Marc Randall (Leeds, UK), Wayne Sunman (Nottingham, UK), Kailash Krishnan (Nottingham, UK)

Neuroimaging Adjudicators

Rob Dineen (Nottingham, UK), Alessandro Adami (Verona, Italy), Lesley Cala (Perth, Australia), Ana Casado (Edinburgh, UK), Rebecca Gallagher (Derby, UK), David Swienton (Leicester, UK), Satheesh Ramalingam (Birmingham, UK)

Platelet substudy

Stan Heptinstall, Sue Fox, Jane May (Nottingham, UK)

Trial Management Committee

Senior Trial Managers: Sally Utton (2012-14), Diane Havard (2015-17)
Trial Manager: Hayley Foster (2013-16)
UK Coordinators: Margaret Adrian (2009-17), Tanya Payne (2009-15), Alice Durham (2013-14), Harriet Howard (2014-15), Michael Stringer (2014-17), Jamie Longmate (2015-17)
International Coordinators: Sharon Ellender (2009-13), Alice Durham (2013-14), Sarah Grant (2013-14), Joanne Keeling (2014-16)
Outcome Coordinators: Sharon Ellender (2009-13), Lynn Stokes (2010-12), Patrick Cox (2011-13), Judith Clarke (2012), Lyndsey Cobane (2012-14), Kathy Whittamore (2013-14), Joanne Keeling (2013-16), Jennifer Smithson (2014-16), James Kirby (2015-16), Gemma Walker (2015-16)
Statisticians: Michael Tracy (2009-10), Cheryl Renton (2009-12), Cyrille Correia (2011), Lydia Fox (2012-13), Aimee Houlton (2012-13), Katie Flaherty (2013-17), Polly Scutt (2013-17), Lisa J Woodhouse (2013-17)
Programming/database management: Graham Watson (2009-11), Liz Walker (2009-17), Richard Dooley (2013-17)
Physicians: Tim England (2009), Chamilla Geeganage (2009-12), Sandeep Ankolekar (2009-11), Kailash Krishnan (2012-15), Jason Appleton (2015-17)
Data managers: Lida Kaur (2009-10), Tanya Jones (2010-13), Clare Randall (2011-12), Dawn Hazle (2012-16), Mark Sampson (2013-17)
Finance: Wim Clarke (2009-17)
Secretaries: Marilyn Stonham (2011), Susan Blencowe (2012-13), Yvonne Smallwood (2012-17), Lauren Dunn (2013-14), Monika Kowalczyk (2014-16), Patricia Robinson (2015-16)
Administrators (temporary): Sarah Bull (2013), Georgina Phillips (2013), Harry Banks (2013-14), Esther Akanya (2014), Sean McLoughlin (2015), Richard Barks (2015-17)

Participating Countries, Sites and Investigators

Denmark

Co-ordinating Centre: H Christensen (National Co-ordinator), L M Christensen (Follow-up Investigator)
Copenhagen University Hospital, Bispebjerg (33): H Christensen, L Bentsen, L M Christensen, C Krarup Hansen, T T Thomsen

Herlev Hospital (91): C Kruuse, H H Jensen, S S Hansen, V Petrovic

Georgia

Co-ordinating Centre: M Beridze (National Co-ordinator), N Beridze (Follow-up Investigator)

Tblisi State Medical Institute (15): N Kakabadze, M Beridze, N Beridze, T Kherkheulidze, D Kakabadze

Khechinashvili Medical University Hospital (46): I Toidze, N Beridze, N Lobjanidze, N Akiashvili

Tblisi Central Hospital (22): A Tevdoradze, N Beridze, T Kherkheulidze, N Khizanishvili, T Tsanava

New Zealand

Co-ordinating Centre: A Ranta (National Co-ordinator), R Taylor (Follow-up Investigator)

Palmerston North Hospital (7): I Iniesta, J Kok, J Duignan, M Funnell, A Ranta, P Cariga, M Rodriguez, R Taylor, I J Watson, S Tennant

United Kingdom

Aberdeen Royal Infirmary (58): M Macleod, J Furnace, H Gow, J Irvine, A Joyson, S Nelson, V Taylor

Airedale NHS Foundation Trust (1): M Smith, R Bellfield, B Hairsine

Arrowe Park Hospital (4): R Davies, A Dodd

Atnagelvin Hospital, Londonderry (1): J Corrigan, M Doherty

Barnsley District General Hospital (39): A Ahmed, C Denniss, S Johnson-Holland, K A Kay

Barts Health NHS Trust - Royal London Hospital (39): R Icart Palau, G Auld, P Daboo, R Erande, G Grimwood, D Hove, L Howaniec, O Redjep

Basildon University Hospital (2): R Rangasamay, G Butt

Birmingham Heartlands Hospital (3): D Sandler, J Reddan, S Stafford

Blackpool Victoria Hospital (1): J McIlmoyle

Bradford Royal Infirmary (4): S Maguire, R Bellfield

Bristol Royal Infirmary (5): P Murphy, J Chambers, L Guthrie, M Osborn, A Steele

Buckinghamshire Healthcare NHS Trust, High Wycombe (31): M Burn, A Benford, A Misra, D Hilton

Cambridge Univ. Hospitals NHS Foundation Trust (8): E O'Brien, E Amis, S Finlay, J Mitchell

Charing Cross Hospital, Imperial (32): O Geraghty, K Harvey, B Hazel, S Mashate, P Wilding

Chesterfield Royal Hospital NHS Foundation Trust (25): M Sajid, M Ball, R Gascoyne

Colchester General Hospital (44): R Sivakumar, A Wright

Countess of Chester NHS Foundation Trust (73): K Chatterjee, S Booth, H Eccleson, C Kelly, S Leason, C Perkins

County Durham and Darlington NHSFT (34): D Bruce, E Brown, S Clayton, M Garside, G Rogers

Croydon University Hospital (10): E Lawrence, S Mahmood, C Watchurst

Doncaster & Bassetlaw Hosps. NHS Foundation Trust (29): D Chadha, L Glover, L Holford, K Smith, D Walstow

Dorset County Hospital NHS Foundation Trust, Dorchester (1): R Williams, L O’Shea, J Goodsell

Eastbourne General Hospital (40): C Athulathmudali, E Barbon

Fairfield General Hospital (7): R Namushi, P Jacob, L Johnson, D Morse

Forth Valley Royal Hospital (11): M Macleod, C McGhee

Frimley Park Hospital NHS Foundation Trust (5): O Speirs, S Atkinson, A Peacocke

Glasgow Royal Infirmary (19): P Langhorne, R Graham, F Wright, C McAlpine

Good Hope Hospital (3): A Ravindrane, J Reddan, S Stafford

Great Western Hospitals NHS Trust (4): M Bajoriene, L Matter, S Windebank

Hampshire Hospitals NHS Foundation Trust (24): E Giallombardo, D Dellafera, C Eglinton, J Wilson

James Cooke University Hospital (46): D Broughton, K Chapman, L Dixon

James Paget University Hospital NHS Foundn. Trust (1): M Zaidi

Kettering General Hospital (1): K Ayes, J Kessell

King's College Hospital (164): D Manawadu, O Adegbaju, J Aeron-Thomas, K Anderson, A Brigden, E Cattermole, J Good, S Hassan, E Khoromana, L Lee-Carbon, K Marks, E Mckenzie, N Sikondari

Kings Mill Hospital (47): M Cooper, K Whysall, I Wynter

Leeds General Infirmary (48): J Bamford, A Hassan, P Wanklyn, M Kambafwile, L Makawa, M Randall, D Waugh, E Veraque

Lincoln County Hospital (14): MGG Soliman, S Arif, R Brown, S Butler, C Hewitt, J Hindle

Lister Hospital (20): A Pusalkar, H Beadle, K Chan, M Siddiqui, P Dangri, S Buddha, A AsokanathanLUH NHS Trust (Leicester Royal Infirmary) (33): A Mistri, D Eveson, K Musarrat, L Manning, S Anand, P Christian, S Khan, C Patel

Macclesfield District General Hospital (16): M Sein, J Banns, E Gibson, T Gordon, Y Gruenbeck, S Wong

Mid Yorkshire Hospitals NHS Trust (Dewsbury & Pinderfields) (61): P Datta, G Bateman, L Jackson, A Needle

Milton Keynes Hospital NHS Foundation Trust (4): Y Duodu, R Oliver, C Padilla-Harris

Monklands Hospital (48): M Barber, D Esson, F Brodie, C McInnes

New Cross Hospital, Wolverhampton (25): K Fotherby, D Butler, D Morgan, K Preece, A Willberry

North Devon District Hospital (2): M Dent, F Hammonds, J Hunt, C Vernon

Northampton General Hospital NHS Trust (8): D O'Kane, F Faola, P Lai, J O'Callaghan, C Smith

Northumbria Specialist Emergency Care Hospital (23): C Price, R Lakey, V Riddell, A Smith, G Storey

Nottingham University Hospitals (220): S Munshi, P Bath, N Sprigg, A Buck, J Clarke, N Gilzeane, M Godfrey, F Hammonds, R Keshvara, C Richardson, J Roffe, L Ryan, F Shelton, W Sunman, A Tittle, J Tomlinson, K Whittamore, G Wilkes

Peterborough & Stamford Hosps NHS Foundation Trust (7): P Owusu-Agyei, N Temple

Pilgrim Hospital (34): D Mangion, A Hardwick, K Netherton

Plymouth Hospitals NHS Trust (15): A Mohd Nor, C Eglinton, B Hyams, S Norman, N Persad

Poole Hospital NHS Foundation Trust (15): S Ragab, C Dickson, J Dube, E Jinks, K Knops, B Wadams

Princess Royal Hospital, Haywards Heath (4): K Ali, J Gaylard, G Spurling

Princess Royal University Hospital (39): L Sztriha, T Ajao, M Alao, F K Chan, P Webster

Queen Alexandra Hospital, Portsmouth (2): P Howard, T J Dobson, L Hyatt

Queen Elizabeth Birmingham (12): D Sims, J Cunningham

Queen Elizabeth Hospital (Gateshead) (37): B Esisi, T Cassidy, M Bokhari, B McClelland, B Mokoena

Queen Elizabeth, the Queen Mother Hospital (51): G Gunathilagan, S Jones, M Reader, G Thomas, S Tilby

Raigmore Hospital (37): P Findlay, F Barrett, F Leslie, S Ross, I Shread

Rotherham District General Hospital (14): J Okwera, J Howe

Royal Cornwall Hospitals NHS Trust (19): F Harrington, G Courtauld, C Schofield

Royal Derby Hospital (69): T England, R Donnelly, M Maddula, J Scott, J Beavan, K Muhidden, I Memon, J Clarke, M Clarke, A Hedstrom, L Mills

Royal Devon & Exeter NHS Foundation Trust (63): A Hemsley, A Bowring, L Boxall, H Kingwell, S Keenan, C Roughan

Royal Liverpool University NHS Trust (69): A Manoj, P Cox, G Fletcher, P Lopez

Royal Preston Hospital (39): H Emsley, B Gregary, A McLoughlin, S Raj

Royal Stoke University Hospital (216): C Roffe, N Abano, A Barry, A Butler, R Carpio, K Castro, K Finney, S Gomm, J Hiden, J Grocott, S Lyjko, H Maguire, A Remegoso, R Sanyal, S Stevens, I Natarajan, J Chembala, G Muddegowda, A

Warusevitane

Royal Surrey County Hospital NHS Trust (13): A Blight, O Balazikova, C Lawlor

Royal United Hospital Bath NHS Trust (25): L Shaw, D Button, D Howcroft, S Lucas, B Madigan, S McCann

Royal Victoria Infirmary, Newcastle-upon-Tyne (53): A Dixit, A Barkat, J Davis, M Fawcett, L Finlay, H Guy, C Hays, V Hogg, E Horsley, C Hubbuck, C Pringle, C Stevenson, K Storey, T Thompson, S Woodward

Russells Hall Hospital (12): A Banerjee, C Allcock, S Merotra

Salford Royal NHS Foundation Trust, Salford Royal (10): C Douglass, E Campbell, R Jarapa, M Johnes, C Keaveney, T Marsden, Z Naing, J Perez, K Shaw

Salisbury District Hospital (15): T Black, A Anthony, C Clarke

Scarborough General Hospital (9): J Paterson, K Deighton, E Temlett

Sheffield Teaching Hospitals NHS Foundation Trust (41): C Blank, C Doyle, S Duty, K Gill, K Harkness, J Howe, C Kamara, E Richards

Solihull Hospital (2): K Elfandi, S Stafford

Southend University Hospital NHS Foundation Trust (9): P Guyler, P Harman, C Khuoge, S Kunhunny, S Tysoe

St Georges Healthcare NHS Trust (183): B Moynihan, T Adedoyin, N Chopra, N Dayal, R Ghatala, N Jeyaraj, I Jones, F Kennedy, L Kerin, N Khanom, S Lewis, S Maheswaran, L Montague, M Niemierko, J O'Reilly, S Trippier, C Watchurst, F Watson

St Peters Hospital, Chertsey (9): P Wilkinson, E Young

Stepping Hill Hospital (11): K Dizayee, H Cochrane, D Morse

Sunderland Royal Hospital (9): J O'Connell, L Mokoena, E Osborne, A Smith

The Calderdale Royal Hospital (41): A Nair, J Greig

The County Hospital, Hereford (8): C Jenkins, J Powell, F Price

The Ipswich Hospital NHS Trust (48): M Chowdhury, S Brixey, L Hunt, N Rands, G Rose, S Stoddart

The Princess Royal Hospital, Telford (2): M Srinivasan, N Motherwell

The Queen Elizabeth Hospital, King’s Lynn (27): R Shekhar, T Fuller, A Lankester, P Lingwood, C Rankin, H Webb

The Royal Bournemouth Hospital (19): B Jupp, J Bell, G Hann, B Longland, C Ovington

Torbay District Hospital (49): B Bhaskaran, G Ayres, C Bailey, H Bearne, J Buxton, P Fitzell, C Hilaire, D Kelly, S Szabo, D Tomlin

Univ Hosp of South Manchester (11): E Gamble, B Charles

University Hospital Aintree (52): R Kumar, T Fluskey, Z Mellor, J Peters, V Sutton

University Hospital Coventry (11): A Kenton, I Martin, S Nyabadza

University Hospital of Ayr (3): S Ghosh, M Henry

University Hospital of North Tees (47): B Kumar, D Bruce, C Ambulo, S Crawford, T Nozedar, M Platton

Victoria Hospital / Queen Margaret Hospital (27): V Cvoro, M Couser, K McCormick, D Wilkinson

Walsall Manor Hospital (6): K Javaid, S Hurdowar

Watford General Hospital (6): T Attygalle, S Sundayi

West Cumberland Hospital (15): O Orugun, H Crowther, R Jolly, U Poultney

West Suffolk NHS Hospital Trust (3): A Azim, M Krasinska-Chavez, J White

Western Sussex Hospitals (6): N Sengupta, J Margalef, M G Metiu

Whiston Hospital (25): S Meenakshisundaram, S Dealing

William Harvey Hospital (44): D Hargroves, E Beranova, L Cowie, H Rudenko, A Thomson, A Verrion

Yeovil District Hospital NHS Foundation Trust (24): K Rashed, S Board, C Buckley, D Hayward, K Jenkins, E Keeling, R Rowland-Axe, C Vickers, D Wood

Repatriation Sites:

Homerton University Hospital (11 repatriated patients): A Lehman, O Redjep, R Erande, G Grimwood, D Hove

Lewisham Healthcare NHS Trust (4 repatriated patients): M Patel, H Russell

Royal Albert Edwards Infirmary, Wigan (3 repatriated patients): H Rehman, D Forrest, P Farren

METHODS

Training of Investigators

All TARDIS investigators were trained in the protocol, Good Clinical Practice and use of the National Institutes of Health Stroke Scale, modified Rankin Scale (mRS) and Barthel Index. Additionally, telephone outcome assessors were trained in and then tested with case scenarios for the mRS.

Schedule for Monitoring of Sites and Data Integrity

Site monitoring was performed by each National Coordinating Centre (NCC) with the aim of ensuring quality control for the delivery of the protocol, collection of data and adherence with national regulations and ethics. Each recruiting site had a start-up visit for training and at least one monitoring visit; further visits were performed as deemed necessary by the NCC. Monitoring visits confirmed the presence of the participant and their consent, eligibility criteria, selected data critical to the trial (demographics, prescription of interventions and blood pressure) and reported serious adverse events.

Central statistical monitoring of the data was performed according to Buyse *et al* [1] during the trial and prior to locking of the data. Checks included logic and range checks, digit preference, comparison of univariate data between sites and comparison of multiple variable models between countries. The monitoring procedures were compliant with the requirements of the sponsor, the national ethics committees and regulatory authorities in the participating countries and fulfilled Good Clinical Practice requirements.

Inclusion and Exclusion criteria [2]

Inclusion criteria

Adults at high risk of recurrent ischaemic stroke:

1. Age ≥50 years
2. Within 48 hours of ictus (24-48 hours if thrombolysed)
3. TIA with limb weakness and/or dysphasia lasting between 10 minutes and <24 hours with no residual symptoms and presenting with any of the following
    a. ABCD2 score ≥4
    b. Crescendo TIA
    c. Already on dual antiplatelet therapy with aspirin and dipyridamole
    d. Positive neuroimaging evidence to support the new event, ischaemic stroke on magnetic resonance (MR) diffusion imaging
4. Ischaemic non cardioembolic stroke presenting with any of the following:
    a. Ongoing limb weakness of more than one hour duration; and/or
    b. Ongoing dysphasia of more than hour duration; and/or
    c. Resolved limb weakness of more than one hour duration with ongoing facial weakness; and/or
    d. Ongoing isolated hemianopia of more than 1 hour duration with positive neuroimaging evidence to support the new event (e.g. ischaemic stroke in the occipital lobe); and/or
    e. Limb weakness that resolves between 24-48 hours after onset; and/or
    f. Dysphasia that resolves between 24-48 hours after onset; and/or
    g. Positive neuroimaging to support the new ischaemic event with MR diffusion.
    h. Already on combined dual antiplatelet therapy (aspirin + dipyridamole)

Notes:

1. Patients who are on monotherapy e.g. aspirin alone, clopidogrel alone or dipyridamole alone, are eligible for recruitment. Similarly, patients who are on combined therapy aspirin and dipyridamole, are eligible for recruitment if they fulfil the above criteria.
2. Patients with posterior fossa events are eligible if they fulfilled the above criteria.
3. Neuroimaging is not necessary for transient ischaemic attack.
4. Crescendo TIA is >1 TIA in one week, with the onset time of the last TIA taken as the time of ictus.
5. Neuroimaging showing a new lesion is required for isolated hemianopia since visual symptoms are common and often vague, and may not be related to stroke.
6. Neuroimaging is essential for ischaemic stroke to exclude intracranial haemorrhage and a non-stroke diagnosis. If the patient received thrombolysis, a post-thrombolysis/pre- TARDIS scan neededs to be done to exclude new thrombolysis associated bleeding prior to enrolment. Typically, this is done routinely as ‘standard of care’, but if it is not done, then it must be done prior to enrolment.
7. Patients thrombolysed for stroke with full recovery in less than 24 hours from the onset of symptoms are eligible for inclusion providing neuroimaging post thrombolysis excluded intracranial haemorrhage.
8. Informed consent from participant. If the participant is unable to give meaningful consent e.g. due to dysphasia, confusion or reduced conscious level, proxy consent may be obtained from a relative, carer or legal representative.

Exclusion criteria

1. Age<50
2. Isolated sensory symptoms or vertigo/dizziness or facial weakness
3. Isolated hemianopia without positive neuroimaging evidence
4. Intracranial haemorrhage
5. Baseline neuroimaging showing parenchymal haemorrhagic transformation (PH I/II) of infarct, subarachnoid haemorrhage or other non ischaemic cause for symptoms
6. Presumed cardioembolic stroke (e.g. history or current atrial fibrillation [AF], myocardial infarction [MI] within 3 months)
7. Participants with contraindications to, or intolerance of, aspirin, clopidogrel or dipyridamole.
8. Participants with definite need for treatment with aspirin, clopidogrel or dipyridamole individually or in combination (e.g. aspirin and clopidogrel for recent MI/acute coronary syndrome)
9. Definite need for full dose oral (e.g. warfarin, dabigatran) or medium to high dose parenteral (e.g. heparin) anti-coagulation. NB Low dose heparin for deep vein thrombosis (DVT) prophylaxis is allowed
10. Definite need for glycoprotein IIb-IIIa inhibitors
11. Patients who have received thrombolysis within 24 hours
12. No enteral access
13. Pre-morbid dependency (mRS>2).
14. Severe high blood pressure (BP) (BP>185/110 mmHg).
15. Haemoglobin less than 10g/dL
16. Platelet count more than 600 x 10^9/L or less than 100 x 10^9/L
17. White cell count more than 30 x 10^9/L or less than 3.5 x 10^9/L
18. Major bleeding within 1 year (e.g. peptic ulcer, intracerebral haemorrhage).
19. Planned surgery during 3 month follow-up (e.g. carotid endarterectomy)

20. Concomitant STEMI or NSTEMI.
21. Stroke secondary to a procedure (e.g. carotid or coronary intervention)
22. Coma (Glasgow Coma Scale, GCS<8)
23. Non-stroke life expectancy<6 months
24. Dementia
25. Participation in another drug or devices trial concurrently or within 30 days. (participants may take part in observational studies or non-drug or devices trials)
26. Geographical or other factors that may interfere with follow-up e.g. no fixed address or telephone contact number, not registered with a GP or overseas visitor.
27. Females of childbearing potential, pregnancy or breastfeeding
28. Patients who have not had post thrombolysis neuroimaging.
29. Patients on aspirin and clopidogrel prior to the underlying event.

Baseline assessments

Age, sex and ethnicity were recorded at baseline. The severity of index stroke was assessed with the National Institutes of Health Stroke Scale (NIHSS, scores range from 0 to 42 with higher scores indicating a more severe neurological deficit [3]) and risk of recurrence after index TIA assessed using the ABCD2 scale (scores range from 0 to 7 with higher scores indicating a higher risk of recurrence [4]). Clinical syndrome was assessed using the Oxfordshire Community Stroke Project classification.[5] Information on prior antiplatelet therapy (none, aspirin, clopidogrel, dipyridamole, other) was also recorded at baseline.

Randomisation

Patients randomised to the guideline group received either combined aspirin (A) and dipyridamole (D), or clopidogrel (C) alone, according to local policy and guidelines, and antiplatelets taken prior to randomisation:

- Each site chose what comparator(s) they wished to use for ischaemic stroke and TIA separately. They could elect to use one regimen only, or randomise between the compactors. The principal investigator could change the choice of comparison group(s) via the database at any stage during the trial, but changes took 48 hours to take effect to avoid changes being made for individual participants.
- To avoid confounding treatment, randomisation was also determined by what antiplatelet(s) had been taken shortly before stroke onset or in hospital prior to randomisation. Because, aspirin is widely used immediately following scanning, it was allowed in all choices prior to randomisation:
    - ACD vs C vs AD - A only before randomisation
    - ACD vs C - A or C only before randomisation, i.e. no D
    - ACD vs AD - A or D only before randomisation, i.e. no C

In the event that the website could not be accessed, participants were randomised by telephoning one of a series of emergency telephone numbers. These participants were randomised without stratification or minimisation.

Procedures

The two approaches for guideline therapy arose because of a change in clinical guidelines. At the start of the trial, the UK National Institute for Health and Care Excellence (NICE) recommended the use of aspirin and dipyridamole for secondary prevention[6] and the initial trial protocol defined this as the guideline comparator. Once clopidogrel became generic, NICE updated their guidance in 2010 with a recommendation that clopidogrel should be used first-line for secondary prophylaxis

after ischaemic stroke (but not TIA due to clopidogrel not being licensed for TIA and the absence of randomised trial data for this indication)[7] and the protocol was updated accordingly. Each site was allowed to choose whether guideline randomisation was to clopidogrel alone, aspirin and dipyridamole or to either comparator; this choice was made separately for stroke and TIA. Choices could be changed but not within 48 hours to prevent them from being adjusted for a particular patient.

At the start of the trial, clopidogrel was available as a branded drug, Plavix. Subsequently, multiple generic versions became available with the advantage of a much lower cost; as a result, recruiting sites increasingly used generic clopidogrel. A number of studies and a meta-analysis have compared Plavix with generic clopidogrel and these do not report any significant difference in antiplatelet responses or effects on vascular outcomes.[8-11]

Outcome measures

The primary outcome comprised a six-level ordered categorical scale:[12] fatal stroke, non-fatal severe stroke (modified Rankin Scale, mRS 4, 5), moderate stroke (mRS 2, 3), mild stroke (mRS 0, 1), TIA, and no stroke or TIA, as published in the Statistical Analysis Plan.[13] The purpose of using an ordinal outcome is twofold. First, it allows the effect of treatment to be assessed on severity of recurrent events as well as their rate. In general, interventions that reduce the risk of recurrence (such as antithrombotics, and blood pressure and lipid lowering therapies) also reduce the severity of those events that do occur;[14,15] similarly, interventions that increase recurrence (e.g. hormone replacement therapy) also increase the severity of events.[16] Second, ordinalising dichotomous events improves statistical power so that sample size can be reduced for a given power, or power increased for a given sample size. The approach can be used with any dichotomous outcome that can encompass a measure of severity, such as recurrence of stroke/TIA, bleeding, and serious adverse events (as done here),[14] Other outcomes such as myocardial infarction, heart failure and venous thromboembolism can similarly be analysed in the same way. Although this approach has been tested empirically using published data,[14] TARDIS is the first trial to use the method prospectively.

Originally, the intention was to analyse across 9 levels of severity comprising the individual seven levels of mRS, TIA and no event (protocol version 1.1, 17/10/2008; protocol version 1.2, 20/05/2009). Subsequently the scale was reduced to five-levels; fatal stroke, moderate-sever stroke (mRS 2-5), mild stroke (mRS 0-1), TIA and no event (protocol version 1.3, 20/12/2011; protocol version 1.4, 26/02/2013; protocol version 1.5, 28/02/2014). Finally, the sale was increased to six levels.[13] The changes resulted from multiple discussions among the statistical and clinical teams on simplifying the scale (from 9 levels) but not over-merging mRS levels, in particular keeping mild, moderate and severe stroke separate. These changes occurred during enrolment and were made blinded to any unblended data analysis. A post hoc analysis comparing these three approaches is given below.

Definition of Events

See Statistical Analysis Plan.[2]

Neuroimaging Scan Adjudication

CT or MRI brain scans were performed according to local site practice at baseline in all patients with ischaemic stroke to confirm the diagnosis; investigators decided whether to perform neuroimaging in patients with TIA according to clinical need. Sites could also perform follow-up scans at any time point after enrolment according to clinical need, e.g. if a neurovascular outcome or haemorrhage was suspected. For this publication, scan-derived information is based on radiological reporting from local sites.

Future reports will utilise information based on central adjudication of neuroimages. Neuroimages were submitted to the International Coordinating Centre in Nottingham using one of two methods:

a) Uploaded onto the trial website as uncompressed encrypted non-anonymised digital DICOM files. Once the trial system had validated the files against the expected patient details, the files were then anonymised.
b) Sent by courier on a CD-ROM or DVD, with files in DICOM format with pseudo-anonymisation of patient details; the patients was identified with their unique study number and initials.

When reviewed, some images were in non-DICOM format (e.g. .PNG, .JPG) and these were converted to DICOM. The anonymised image files were then presented to a panel of expert adjudicators using a browser-based system driven from the trial database. Adjudicators were trained and assessed using the ACCESS system (*www.neuroimage.co.uk/sirs*)[17,18] and reviewed scans blinded to treatment assignment. Adjudication parameters were derived from the IST-3 and ENOS trial image adjudication systems[19,20] and included information on:

a) Presence of an acute stroke lesion: location, mass effect and presence of secondary ischaemia or haemorrhage.
b) Presence of pre-stroke changes: atrophy, white matter hyperintensities, old stroke.

Information from adjudication was used to inform the final diagnosis for all participants with a received scan; where clinical and radiological information were incongruent, RD performed a second adjudication to confirm imaging findings.

Independent Data Monitoring Committee (DMC)

An independent DMC reviewed unblinded data in confidence every 6 months; altogether they met on 13 occasions and recommended trial continuation for all but the last data review. The DMC was responsible for safeguarding the interests of trial participants, assessing the safety and efficacy of the intervention during the trial, assessing data integrity and for monitoring the overall conduct of the trial. The DMC reviewed the recruitment of participants and assessed safety and efficacy measures by treatment group. Data were reviewed twice yearly throughout the recruitment period of the trial.

The DMC followed a pre-defined charter and were charged with informing the Trial Steering Committee if, at any time, the data showed evidence beyond reasonable doubt of a difference between the randomised groups in the primary outcome. They also considered these data in the light of external information such as results from completed trials. One interim analysis was performed; additionally, the DMC could perform statistical comparisons as they deemed necessary.

The DMC were given specific stopping rules for efficacy and hazard but not futility; stopping criteria were based on the Haybittle-Peto rule (i.e. a difference of 3 standard errors was to be considered as clear evidence of a treatment effect):

"The balance between safety and efficacy should be considered.

With respect to safety the following outcomes in particular will initiate discussion and minuting of detailed reasons for recommending early stopping or continuation of the study:

- The primary outcome ('shift' in modified Rankin Scale in participants having a recurrent stroke event or TIA) favours the control group (who receive standard antiplatelet therapy but not clopidogrel), P<0.01 (nominal, 2-sided).
- Combined outcome of fatal or non-fatal stroke or major bleeding favours the control group, P< 0.01 (nominal, 2-sided).
- The overall rate of symptomatic intracranial haemorrhage exceeds 2%.
- During the start-up phase, major bleeding favours the control group, P< 0.01 (nominal, 2-sided).

In making any decision, the committee will consider the overall internal and external evidence, the multiplicity of testing and the possibility that the trends in the data might be reversed with longer follow-up or increased recruitment.

With respect to efficacy, the committee will conduct formal interim analyses, after 40% and 70% of the target number of

participants have been enrolled and had their 90 day outcome assessed, based on the following outcome.

- Combined outcome of fatal or non-fatal stroke or major bleeding event favours the clopidogrel group, P < 0.001 (2-sided).

In making any decision, the committee will consider the overall internal and external evidence."

RESULTS

Bleeding by time to treatment

The risk of haemorrhage for intensive and guideline antiplatelets diverged from the start of randomised treatment (Figure S5); significantly more, and more severe, bleeding was apparent not just at day 90 (Table 3) but also by days 14 and 21 (Table S2).

Antiplatelet treatment and adherence

In 3096 recruited participants, 1556 were randomised to the intensive antiplatelet group and 1540 to the guideline group; of the latter more participants were randomised to clopidogrel alone (849, 55.1%) than combined aspirin and dipyridamole (691, 44.9%). Adherence to randomised treatment over the first 7 days was fair in both treatment groups: initial treatment was received in 83.5% of patients and 98.4% received at least some randomised treatment during the first week (Table S4). Participants randomised to intensive antiplatelets were more likely to receive all treatment in the first week than those randomised to guideline treatment.

The rate of treatment crossover was low: 6 (0.4%) participants randomised to the intensive group did not receive this and 30 (1.9%) participants randomised to the guideline group received intensive antiplatelets at some point in the treatment phase. During treatment, 37 (1.2%) participants had carotid endarterectomy leading to temporary cessation of one or more antiplatelets (Table S1). A further 31 (1.0%) participants had antiplatelet therapy replaced with oral anticoagulation following identification of atrial fibrillation. Gastroprotection was provided in 803 (25.9%) patients. Following the final DMC review and early closure to recruitment, participants randomised to intensive antiplatelet therapy were asked immediately to switch treatment to guideline therapy.

Ordinal outcome

Originally the trial intended to assess the effect of intervention on a nine-level ordinal outcome for stroke and TIA, and subsequently a five-level outcome. However, these were felt to be over- or under-granular respectively, and the six-level outcome was chosen for the final analysis. Decisions on these changes were made blinded to treatment assignment. In *post hoc* analyses, the original outcomes were analysed and gave comparable results (Table S5).

Treatment by subgroup interactions

The statistical analysis plan detailed treatment by severity interactions for both ordinal stroke and major bleeding and these are shown here in Figure S1 and Figure S4 respectively. Subsequently, the reviewers of this publication suggested alternative cut points for the measures of severity, onset to randomisation, and degree of carotid stenosis. These *post hoc* analyses, and the original results, are shown in Table S6. All the interaction tests remain non-significant.

DISCUSSION

Additional comment

The assessment of intensive treatment, based on the combination of three individually effective antiplatelet drugs, was a logical extension of previous observations in patients with acute cerebral ischaemia; these showed that aspirin is superior to no antiplatelet in preventing early recurrence [21,22] and that dual therapy might be superior to mono-therapy.[23] The CHANCE trial demonstrated that combined aspirin and clopidogrel was superior to aspirin alone in preventing recurrence at day 90 in Chinese patients.[24] The ongoing Platelet-oriented inhibition in new TIA and minor ischemic stroke (POINT) trial is assessing this question in Western populations.[25] The findings in TARDIS are incongruent with those in the PRoFESS megatrial (which studied long term antiplatelet therapy) where similar rates of recurrent stroke were present for combined aspirin and dipyridamole vs. clopidogrel alone.[26]

REFERENCES

1. Buyse M, George SL, Evans S, et al. The role of biostatistics in the prevention, detection and treatment of fraud in clinical trials. *Stat Med* 1999; 18(24): 3435-51.

2. Bath P, Robson K, Woodhouse L, et al. Statistical analysis plan for the 'Triple Antiplatelets for Reducing Dependency after Ischaemic Stroke' (TARDIS) trial. *International journal of stroke* 2015; 10(3): 449-51.

3. Adams HP, Davis PH, Leira EC, et al. Baseline NIH Stroke Scale score strongly predicts outcome after stroke. A report of the trial of Org 10172 in Acute Stroke Treatment (TOAST). *Neurology* 1999; 53: 126-31.

4. Johnston SC, Rothwell PM, Nguyen-Huynh MN, et al. Validation and refinement of scores to predict very early stroke risk after transient ischaemic attack. *Lancet* 2007; 369(9558): 283-92.

5. Bamford J, Sandercock P, Dennis M, Burn J, Warlow C. Classification and natural history of clinically identifiable subtypes of cerebral infarction. *Lancet* 1991; 337(8756): 1521-6.

6. National Institute for Health and Clincal Excellence (NICE). Clopidogrel and modified release dipyridamole in the prevention of occlusive vascular events. National Institute for Health and Clincal Excellence; 2005.

7. NICE. Clopidogrel and modified release dipyridamole for the prevention of occlusive vascular events. 2010; (December 2010).

8. Khosravi AR, Pourmoghadas M, Ostovan M, et al. The impact of generic form of Clopidogrel on cardiovascular events in patients with coronary artery stent: results of the OPCES study. *J Res Med Sci* 2011; 16(5): 640-50.

9. Sambu N, Radhakrishnan A, Curzen N. A Randomized Crossover Study Comparing the Antiplatelet Effect of Plavix Versus Generic Clopidogrel. *Journal of Cardiovascular Pharmacology* 2012; 60(6): 495-501.

10. Caldeira D, Fernandes RM, Costa J, David C, Sampaio C, Ferreira JJ. Branded versus generic clopidogrel in cardiovascular diseases: a systematic review. *J Cardiovasc Pharmacol* 2013; 61(4): 277-82.

11. McGregor GP. Pivotal Bioequivalence Study of Clopacin®, a Generic Formulation of Clopidogrel 75 mg Film-Coated Tablets. *Adv Ther* 2016; 33(2): 186-98.

12. Bath PMW, Geeganage CM, Gray LJ, Collier T, Pocock S. Use of ordinal outcomes in vascular prevention trials: comparison with binary outcomes in published stroke trials. *Stroke* 2008; 39(10): 2817-23.

13. Bath P, Robson K, Woodhouse L, et al. Statistical analysis plan for the 'Triple Antiplatelets for Reducing Dependency after Ischaemic Stroke' (TARDIS) trial. *International Journal of Stroke* 2015; 10(3): 449-51.

14. Bath PMW, Geeganage C, Gray LJ, Collier T, Pocock S. Use of ordinal outcomes in vascular prevention trials: Comparison with binary outcomes in published trials. *Stroke* 2008; 39(10): 2817-23.

15. Bath PMW, Geeganage CM, Gray LJ. Ordinal reanalysis of the SHEP trial. *Stroke* 2008; 39(9): E145-E.

16. Sare GM, Gray LJ, Bath PM. Association between hormone replacement therapy and subsequent arterial and venous vascular events: a meta analysis. *European Heart Journal* 2008; 29(16): 2031-41.

17. Wardlaw JM, Farrall AJ, Perry D, et al. Factors influencing the detection of early CT signs of cerebral ischemia: an internet-based, international multiobserver study. *Stroke* 2007; 38(4): 1250-6.

18. Wardlaw JM, von Kummer R, Farrall AJ, Chappell FM, Hill M, Perry D. A large web-based observer reliability study of early ischaemic signs on computed tomography. The Acute Cerebral CT Evaluation of Stroke Study (ACCESS). *PLoS One* 2010; 5(12): e15757.

19. Sandercock P, Wardlaw JM, Lindley RI, et al. The benefits and harms of intravenous thrombolysis with recombinant tissue plasminogen activator within 6 h of acute ischaemic stroke (the third international stroke trial [IST-3]): a randomised controlled trial. *Lancet* 2012; 379(9834): 2352-63.

20. Bath P, Woodhouse L, Scutt P, et al. Efficacy of nitric oxide, with or without continuing antihypertensive treatment, for management of high blood pressure in acute stroke (ENOS): a partial-factorial randomised controlled trial. *The Lancet* 2015; 385(9968): 617-28.

21. International Stroke Trial Collaborative Group. The International Stroke Trial (IST); a randomised trial of aspirin, subcutaneous heparin, both, or neither among 19435 patients with acute ischaemic stroke. *Lancet* 1997; 349: 1569-81.

22. CAST (Chinese Acute Stroke Trial) Collaborative Group. CAST: randomised placebo-controlled trial of early aspirin use in 20,000 patients with acute ischaemic stroke. *Lancet* 1997; 349: 1641-9.

23. Geeganage CM, Diener HC, Algra A, et al. Dual or Mono Antiplatelet Therapy for Patients with Acute Ischemic Stroke or Transient Ischemic Attack: Systematic Review and Meta-Analysis of Randomized Controlled Trials. *Stroke* 2012; 43(4): 1058-66.

24. Wang Y, Wang Y, Zhao X, et al. Clopidogrel with aspirin in acute minor stroke or transient ischemic attack. *N Engl J Med* 2013; 369(1): 11-9.

25. Johnston S, Easton J, Farrant M, et al. Platelet-oriented inhibition in new TIA and minor ischemic stroke (POINT) trial: rationale and design. *Int J Stroke* 2013; 8(6): 479-83.

26. Diener HC, Sacco RL, Yusuf S, et al. Effects of aspirin plus extended-release dipyridamole versus clopidogrel and telmisartan on disability and cognitive function after recurrent stroke in patients with ischaemic stroke in the Prevention Regimen for Effectively Avoiding Second Strokes (PRoFESS) trial: a double-blind, active and placebo-controlled study. *Lancet Neurol* 2008; 7(10): 875-84.

27. Schellinger PD, Bath PMW, Lees K, et al. Assessment of addtional endpoints relevant to the benefit of patients after stroke - what, when, where, in whom. *Int J Stroke* 2012; 7(3): 227-30.
28. Sulter G, Steen C, de Keyser J, 1540. Use of the Barthel Undex and modified Rankin Scale in acute stroke trials. *Stroke* 1999; 30: 1538-41.
29. Zung WWK. A self-rating depression scale. *Archives of General Psychiatry* 1965; 12: 63-70.
30. Roccaforte MD, Burke WJ, Bayer BL, Wengel SP. Validation of a telephone version of the mini-mental state examination. *Journal of the American Geriatric Society* 1992; 40(7): 697-702.
31. Desmond DW, Tatemichi TK, Hanzawa L. The telephone interview for cognitive status (TICS): Reliability and validity in a stroke sample. *International Journal of Geriatric Psychiatry* 1994; 9: 803-7.
32. Tombaugh T, Kozak J, Rees L. Normative data stratified by age and education for two measures of verbal fluency: FAS and animal naming. *Arch Clin Neuropsychol* 1999; 14(2): 167-77.
33. Brooks R, with the EuroQol Group. EuroQol: the current state of play. *Health Policy* 1996; 37: 53-72.

LIST OF SUPPLEMENTARY TABLES AND FIGURES

Tables **Page**

Table S1. Secondary outcomes 18
Table S2. Major bleeding by time from start of treatment 19
Table S3. Serious adverse events (SAEs) 20
Table S4. Adherence to randomised treatment 22
Table S5. Analyses of primary outcome by number of outcome levels 23
Table S6. Treatment by subgroup interactions for recurrence and bleeding 24

Figures

Figure S1. Primary outcome in pre-determined subgroups 26
Figure S2. Kaplan Meier of stroke or TIA 27
Figure S3. Distribution of bleeding by severity 28
Figure S4. Major bleeding in pre-determined subgroups 29
Figure S5. Kaplan Meier plot of major bleeding 30
Figure S6. Kaplan Meier of death 31
Figure S7. Distribution SAEs by severity 32

Table S1. Secondary outcomes.[27]

Outcome	N	Intensive [N=1556]	Guideline [N=1540]	Adjusted MD (95% CI)	P value
Secondary outcomes					
BI (/100) ‡ [28]	2980	92·4 (19·8)	93·2 (18·7)	-0·6 (-1·9, 0·6)	0·30
ZDS (/102·5) ‡ [29]	2506	46·1 (17·0)	46·6 (17·5)	-0·4 (-1·6, 0·9)	0·59
t-MMSE (/23) ‡ [30]	2360	18·3 (4·2)	18·3 (4·4)	0·0 (-0·3, 0·3)	0·94
TICS-M (/37) ‡ [31]	2389	21·1 (6·2)	21·0 (6·5)	0·0 (-0·5, 0·5)	0·93
Verbal fluency ‡ [32]	2412	17·0 (7·6)	17·0 (7·6)	0·0 (-0·5, 0·6)	0·96
EQ-VAS (/100) ‡	2859	72·8 (21·5)	72·0 (22·4)	0·9 (-0·6, 2·5)	0·23
EQ-5D3-HSUV (/1) ‡ [33]	2991	0·7 (0·3)	0·7 (0·3)	0·0 (0·0, 0·0)	0·43

Data are mean (standard deviation) and mean difference with 95% confidence intervals. Comparison by multiple linear regression with adjustment for baseline factors. MD: mean difference; BI: Barthel Index; EQ-5D3-HSUV: EuroQol-5 dimensions 3 levels-health status utility values; EQ-VAS: EuroQol-Visual Analogue Scale; mRS: modified Rankin Scale; t-MMSE: modified telephone-Mini-Mental State Examination; TICS-M: Telephone Interview Cognition Scale-modified; ZDS: Zung Depression Scale

‡: Death = BI -5, ZDS 102.5, t-MMSE -1, TICS-M -1, Verbal fluency -1, EQ-5D-HSUV 0, EQ-VAS -1

Table S2. *Post hoc* analyses of major bleeding by time from start of treatment.

Analysis	Day	Adjusted HR (95% CI)	P value
Post hoc	07	1·49 (0·63, 3·53)	0·37
Post hoc	14	2·48 (1·14, 5·41)	0·023
Post hoc	21	2·30 (1·13, 4·69)	0·021
Post hoc	30	2·71 (1·35, 5·43)	0·0050
Main	90	2·23 (1·25, 3·96)	0·0063

Table S3. Cumulative number of participants with at least one serious adverse event (SAE) by day 35 (end of treatment washout) and day 90 (end of follow-up), by treatment group.

Number (%)	All			Fatal		
Cause	Intensive	Guideline	P value	Intensive	Guideline	P value
By day 7	191 (12·4)	150 (9·8)	0·022	2 (0·1)	4 (0·3)	0·42
By day 35	278 (18·0)	255 (16·7)	0·32	7 (0·5)	10 (0·7)	0·46
By day 90	335 (21·7)	327 (21·4)	0·81	13 (0·8)	22 (1·4)	0·13
By severity						
SAE	335 (21·7)	327 (21·4)	0·60			
Ordinal			0·69			
Fatal	13 (0·8)	22 (1·4)	0·13	13 (0·8)	22 (1·4)	0·13
Severe	54 (3·5)	39 (2·5)	0·12			
Moderate	167 (10·8)	148 (9·7)	0·29			
Mild	101 (6·5)	118 (7·7)	0·21			
None	1208 (78·4)	1204 (78·7)	0·87			
By site						
Neurological	106 (6·9)	84 (5·5)	0·11	3 (0·2)	7 (0·5)	0·22
Initial stroke						
Complication	3 (0·2)	5 (0·3)	0·48	0 (0·0)	3 (0·2)	1·00
Extension	26 (1·7)	20 (1·3)	0·39	1 (0·1)	1 (0·1)	1·00
Cardiac	82 (5·3)	84 (5·5)	0·84	2 (0·1)	2 (0·1)	0·99
Atrial fibrillation	52 (3·4)	64 (4·2)	0·24	0 (0·0)	1 (0·1)	1·00
Failure	5 (0·3)	2 (0·1)	0·28			
Hypertension	3 (0·2)	3 (0·2)	0·99			
Hypotension	4 (0·3)	7 (0·5)	0·37			
Sudden death	2 (0·1)	0 (0·0)	NC	2 (0·1)	0 (0·0)	NC
Gastrointestinal	43 (2·8)	15 (1·0)	0·00042	2 (0·1)	1 (0·1)	0·57
Infarction	-	-	-	-	-	-
Respiratory	32 (2·1)	38 (2·5)	0·45	2 (0·1)	3 (0·2)	0·65
Pneumonia	16 (1·0)	8 (0·5)	0·11	1 (0·1)	2 (0·1)	0·57
PE	5 (0·3)	7 (0·5)	0·56	1 (0·1)	0 (0·0)	NC
Other	131 (8·5)	158 (10·3)	0·083	4 (0·3)	9 (0·6)	0·17
Unattended death	1 (0·1)	0 (0·0)	NC	1 (0·1)	0 (0·0)	NC
DVT	7 (0·5)	4 (0·3)	0·38	-	-	-
Malignancy	8 (0·5)	13 (0·8)	0·27	2 (0·1)	7 (0·5)	0·12
Septicaemia	0 (0·0)	1 (0·1)	NC	0 (0·0)	1 (0·1)	NC
UTI	12 (0·8)	13 (0·8)	0·83	-	-	-
Renal failure	2 (0·1)	2 (0·1)	0·99	-	-	-

Other	11 (0·7)	15 (1·0)	0·42	-	-	-

Data are number (%) of affected participants. Comparison by binary logistic regression, cox proportional hazards models, or ordinal logistic regression.

DVT: deep vein thrombosis; PE: pulmonary embolism; UTI: urinary tract infection

Table S4. Adherence and reasons for non-adherence to randomised treatment during the first 7 days, by treatment group. Data are number (%).

Reasons for non-adherence	All	Intensive	Guideline	P value
Participants randomised	3096	1556	1540	
Adherence during first 7 days				
First treatment	2584 (83·5)	1391 (89·4)	1193 (77·5)	< 0·0001
All treatments	2122 (68·5)	1097 (70·5)	1025 (66·6)	0·030
Any treatment	3045 (98·4)	1525 (98·0)	1520 (98·7)	0·14
Incomplete treatment by day 7 (%)	974 (31·5)	459 (29·5)	515 (33·4)	0·030
Discharged	652 (21·1)	299 (19·2)	353 (22·9)	0·026
Lost to follow-up	51 (1·6)	23 (1·5)	28 (1·8)	0·45
Recurrent stroke/TIA	52 (1·7)	22 (1·4)	30 (1·9)	0·33
Bleeding event	93 (3·0)	57 (3·7)	36 (2·3)	0·032
Adverse event, unacceptable				
Headache	27 (0·9)	21 (1·3)	6 (0·4)	0·0022
Rash	5 (0·2)	3 (0·2)	2 (0·1)	0·65
Serious adverse event	95 (3·1)	50 (3·2)	45 (2·9)	0·63
Death	11 (0·4)	5 (0·3)	6 (0·4)	0·72
Anticoagulation for AF	31 (1·0)	18 (1·2)	13 (0·8)	0·51
Carotid endarterectomy	37 (1·2)	19 (1·2)	18 (1·2)	0·94
Other	185 (6·0)	84 (5·4)	101 (6·6)	0·13

AF: atrial fibrillation; TIA: transient ischaemic attack.

Note: When discharged, participants were given the remaining part of their course of antiplatelet(s) to complete at home.

Table S5. *Post hoc* comparison of analyses of the primary efficacy outcome based on 9, 6 and 5 ordered categorical levels.

Analysis	Levels	Ordinal or binary stroke/TIA	Adjusted cOR/HR (95% CI)	P value
Post hoc	9	mRS 6 / 5 / 4 / 3 / 2 / 1 / 0 / TIA / no event	0·90 (0·67, 1·20)	0·47
Primary	6	mRS 6 / 4-5 / 2-3 / 0-1 / TIA / no event	0·90 (0·67, 1·20)	0·47
Post hoc	5	mRS 6 / 2-5 / 0-1 / TIA / no event	0·92 (0·67, 1·27)	0·61
Sensitivity	2	mRS 0-6 and TIA / no event	0·87 (0·66, 1·16)	0·34

Table S6. *Post hoc* analyses of treatment by stroke severity/TIA, onset to randomisation, and degree of carotid stenosis interactions for ordinal stroke/TIA recurrence and ordinal bleeding. *Post hoc* cut-points suggested by reviewers.

Analysis		Total N	Intensive	Guideline	OR (95% CI)	P	Interaction
Stroke severity							
Stroke/TIA							
Original							0·070
	NIHSS <=3 or TIA	2193	1098 (71·3%)	1095 (71·6%)	0·76 (0·54, 1·06)	0·10	
	NIHSS >3	877	442 (28·7%)	435 (28·4%)	1·41 (0·78, 2·54)	0·26	
Post hoc							0·53
	NIHSS <=5 or TIA	2635	1306 (84·8%)	1329 (86·9%)	0·86 (0·63, 1·17)	0·33	
	NIHSS >5	435	234 (15·2%)	201 (13·1%)	1·16 (0·48, 2·80)	0·75	
Post hoc							0·18
	NIHSS <=3 or TIA	2193	1098 (71·3%)	1095 (71·6%)	0·76 (0·54, 1·06)	0·10	
	NIHSS 4-6	551	263 (17·1%)	288 (18·8%)	1·45 (0·71, 2·98)	0·31	
	NIHSS >6	326	179 (11·6%)	147 (9·6%)	1·38 (0·49, 3·88)	0·55	
Major bleeding							
Original							0·64
	NIHSS <=3 or TIA	2195	1099 (71·3%)	1096 (71·6%)	2·39 (1·86, 3·07)	<0·0001	
	NIHSS >3	877	442 (28·7%)	435 (28·4%)	2·66 (1·77, 4·01)	<0·0001	
Post hoc							0·44
	NIHSS <=5 or TIA	2637	1307 (84·8%)	1330 (86·9%)	2·40 (1·91, 3·02)	<0·0001	
	NIHSS >5	435	234 (15·2%)	201 (13·1%)	3·10 (1·61, 5·96)	0·00071	
Post hoc							0·43
	NIHSS <=3 or TIA	2195	1099 (71·3%)	1096 (71·6%)	2·39 (1·86, 3·07)	<0·0001	
	NIHSS 4-6	551	263 (17·1%)	288 (18·8%)	2·34 (1·44, 3·79)	0·00060	
	NIHSS >6	326	179 (11·6%)	147 (9·6%)	3·92 (1·75, 8·77)	0·00089	
Time							
Stroke/TIA							
Original							0·17
	OTR <=12 hours	310	144 (9·4%)	166 (10·8%)	0·78 (0·37, 1·64)	0·51	
	OTR 12.1-24 hours	645	340 (22·1%)	305 (19·9%)	0·56 (0·30, 1·03)	0·063	
	OTR >24 hours	2115	1056 (68·6%)	1059 (69·2%)	1·09 (0·75, 1·57)	0·65	
Post hoc							0·069
	OTR <=24 hours	955	484 (31·4%)	471 (30·8%)	0·63 (0·39, 1·00)	0·052	
	OTR >24 hours	2115	1056 (68·6%)	1059 (69·2%)	1·09 (0·75, 1·57)	0·65	
Major bleeding							
Original							0·098
	OTR <=12 hours	310	144 (9·3%)	166 (10·8%)	1·24 (0·62, 2·46)	0·54	
	OTR 12.1-24 hours	646	340 (22·1%)	306 (20.0%)	2·26 (1·38, 3·68)	0·0011	
	OTR >24 hours	2116	1057 (68·6%)	1059 (69·2%)	2·77 (2·14, 3·58)	<0·0001	
Post hoc							0·10
	OTR <=24 hours	956	484 (31·4%)	472 (30·8%)	1·86 (1·25, 2·75)	0·0020	
	OTR >24 hours	2116	1057 (68·6%)	1059 (69·2%)	2·77 (2·14, 3·58)	<0·0001	

Carotid stenosis							
Stroke/TIA							
Original							0.70
	<50%	2421	1211 (86.1%)	1210 (85.9%)	0.82 (0.59, 1.15)	0.25	
	>=50%	394	196 (13.9%)	198 (14.1%)	0.96 (0.48, 1.91)	0.90	
Post hoc							0.43
	0%	1375	672 (47.8%)	703 (49.9%)	0.97 (0.61, 1.53)	0.88	
	>0%	1440	735 (52.2%)	705 (50.1%)	0.76 (0.51, 1.13)	0.17	
Major bleeding							
Original							0.25
	<50%	2422	1211 (86.0%)	1211 (85.9%)	2.50 (1.95, 3.20)	<0.0001	
	>=50%	395	197 (14.0%)	198 (14.1%)	1.69 (0.98, 2.91)	0.059	
Post hoc							0.42
	0%	1376	672 (47.7%)	704 (50.0%)	2.61 (1.86, 3.67)	<0.0001	
	>0%	1441	736 (52.3%)	705 (50.0%)	2.13 (1.58, 2.87)	<0.0001	

NIHSS: National institutes of Health Stroke Scale; OTR: onset to randomisation.

Figure S1. Unadjusted common odds ratio for the primary efficacy outcome in pre-specified subgroups.

	N	OR (95% CI)	Interaction p
Graphical Region			0·41
UK	2931	0·9 (0·7, 1·2)	
Other	139	0·4 (0·1, 2·5)	
Age			0·53
<= 75	2195	0·8 (0·6, 1·2)	
> 75	875	1·0 (0·6, 1·7)	
Sex			0·67
Female	1136	0·8 (0·5, 1·3)	
Male	1934	0·9 (0·6, 1·3)	
Index event			0·85
Ischaemic stroke	2126	0·9 (0·6, 1·3)	
TIA	944	0·9 (0·5, 1·3)	
Clinical Syndrome			0·92
LACS	1282	1·0 (0·6, 1·5)	
POCS	211	0·6 (0·2, 2·3)	
PACS	1396	0·8 (0·6, 1·3)	
TACS	179	0·9 (0·3, 2·6)	
NIHSS			0·07
<= 3 or TIA	2193	0·8 (0·5, 1·1)	
> 3	877	1·4 (0·8, 2·5)	
ABCD2 in TIA			0·80
<= 5	612	0·9 (0·5, 1·7)	
> 5	332	0·8 (0·4, 1·6)	
Crescendo TIA			0·89
No	2886	0·9 (0·7, 1·2)	
Yes	184	0·8 (0·4, 1·9)	
No. of antiplatelets at baseline			0·31
0	1995	0·8 (0·5, 1·2)	
1	976	1·1 (0·7, 1·8)	
>=2	99	0·4 (0·1, 1·7)	
Type of randomised comparator			0·067
Aspirin/dipyridamole	1109	0·7 (0·4, 1·1)	
Clopidogrel	1428	1·2 (0·8, 1·9)	
Systolic BP			0·32
<= 140	1361	0·7 (0·4, 1·1)	
140 - 160	1114	1·0 (0·6, 1·6)	
> 160	595	1·2 (0·7, 2·1)	
Time to randomisation			0·17
<= 12	310	0·8 (0·4, 1·6)	
12 - 24	645	0·6 (0·3, 1·0)	
> 24	2115	1·1 (0·8, 1·6)	
Low dose heparin			NC
Yes	6	NC	
No	3064	0·9 (0·7, 1·2)	
Thrombolysis			0·69
Yes	338	1·1 (0·4, 2·6)	
No	2732	0·9 (0·6, 1·2)	
Gastroprotection			0·56
Yes	795	0·8 (0·5, 1·3)	
No	2275	0·9 (0·7, 1·3)	
Small vessel disease			0·83
Yes	1450	0·9 (0·6, 1·3)	
No	1264	0·8 (0·5, 1·3)	
Old vascular lesions			0·44
Yes	1583	0·8 (0·5, 1·2)	
No	1131	1·0 (0·6, 1·6)	
Ipsilateral carotid stenosis			0·7
< 50%	2421	0·8 (0·6, 1·1)	
>= 50%	394	1·0 (0·5, 1·9)	
**All Patients**	**3070**	**0·9 (0·7, 1·2)**	**0·39**

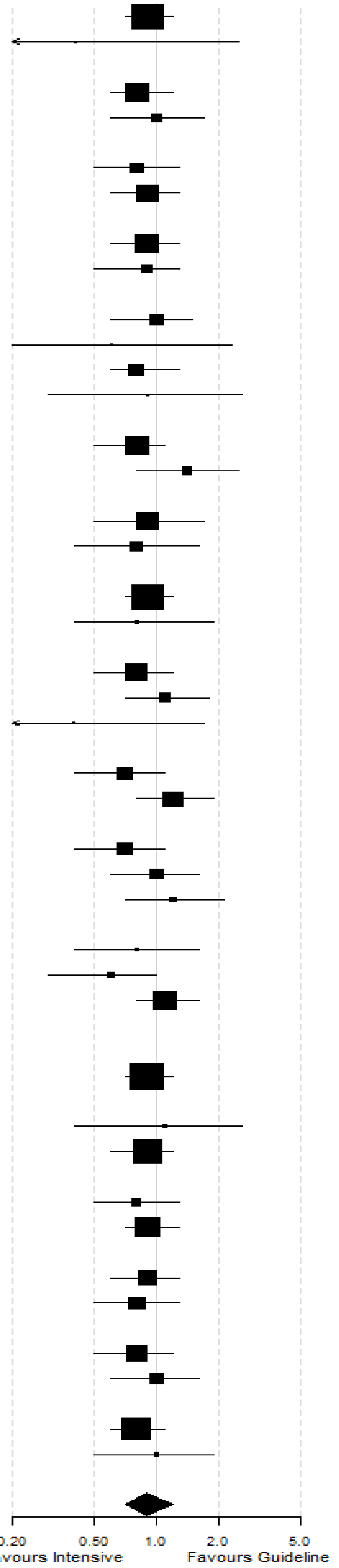

Note: 533 patients who could be randomised to either combined aspirin and dipyridamole, or clopidogrel alone, are excluded for the analysis by Type of randomised comparator.

Figure S2. Kaplan Meier curve for stroke or TIA during the 90 days of follow-up after randomisation.

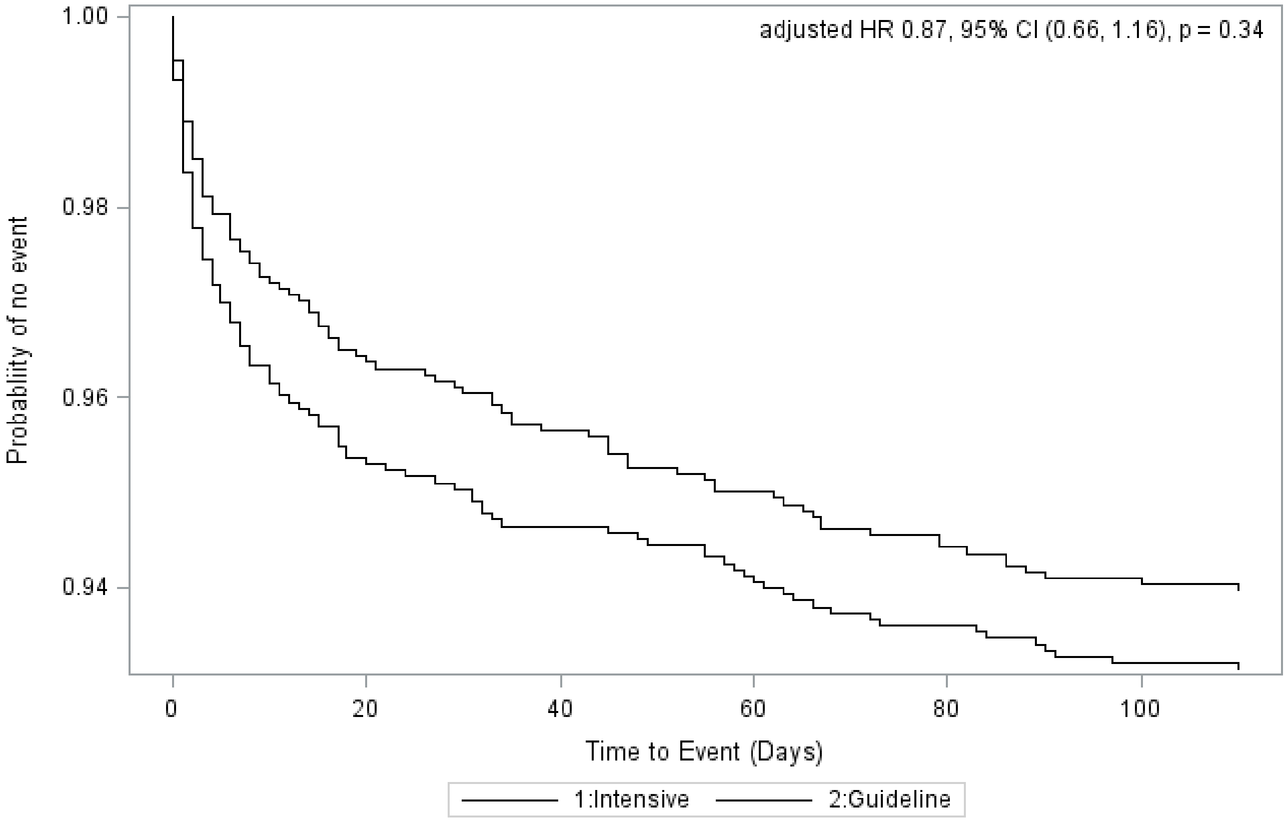

1	1540	1485	1473	1463	1454	1449
2	1530	1459	1448	1440	1432	1426

Figure S3. Distribution of bleeding by severity. Comparison by ordinal logistic regression adjusted for baseline factors.

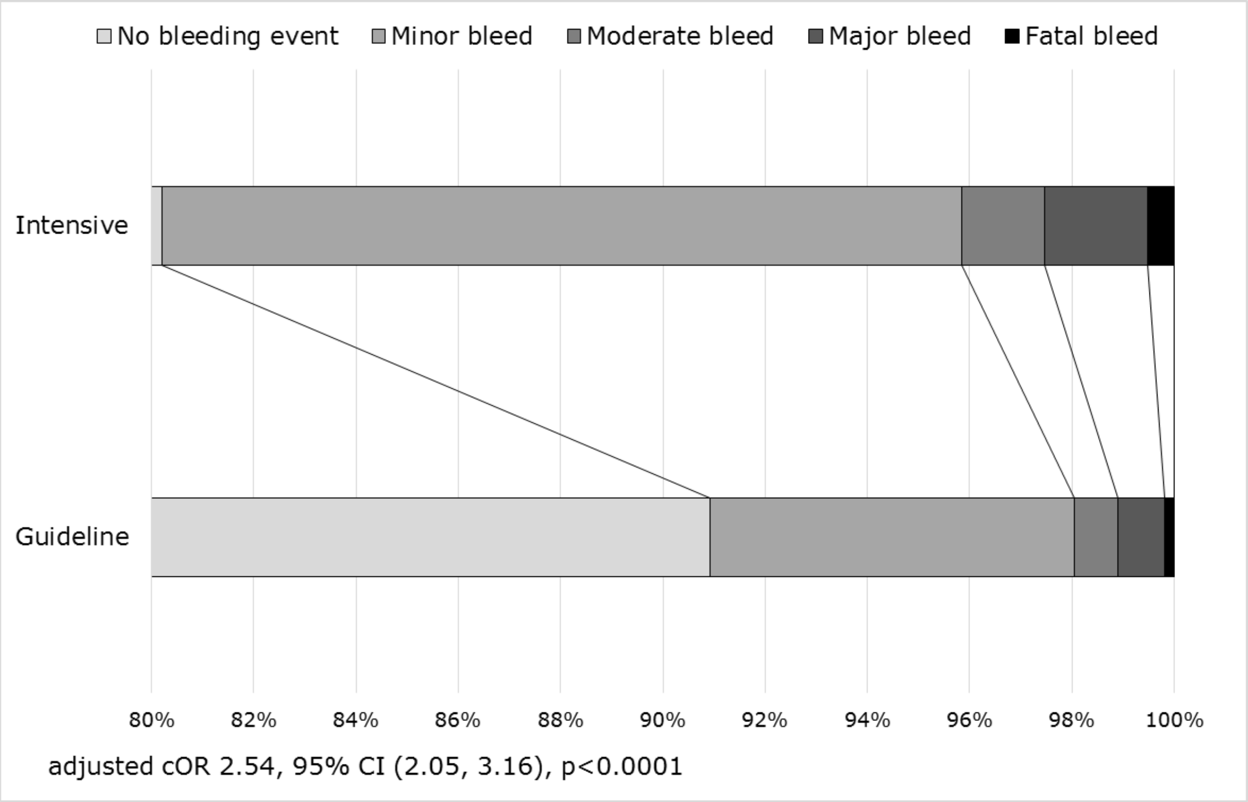

Figure S4. Unadjusted common odds ratio for major bleeding in pre-specified subgroups.

	N	OR (95% CI)	Interaction p
Graphical Region			0·39
UK	2932	2·5 (2·0, 3·1)	
Other	140	1·4 (0·4, 5·1)	
Age			0·23
<= 75	2197	2·7 (2·1, 3·5)	
> 75	875	2·0 (1·4, 3·0)	
Sex			0·66
Female	1138	2·6 (1·9, 3·7)	
Male	1934	2·4 (1·8, 3·1)	
Index event			0·19
Ischaemic stroke	2127	2·7 (2·1, 3·5)	
TIA	945	2·0 (1·3, 2·9)	
Clinical Syndrome			0·84
LACS	1283	2·3 (1·7, 3·2)	
POCS	211	3·3 (1·5, 7·5)	
PACS	1397	2·5 (1·8, 3·4)	
TACS	179	3·0 (1·2, 7·4)	
NIHSS			0·64
<= 3 or TIA	2195	2·4 (1·9, 3·1)	
> 3	877	2·7 (1·8, 4·0)	
ABCD2 in TIA			0·87
<= 5	613	1·9 (1·2, 3·1)	
> 5	332	2·1 (1·1, 4·0)	
Crescendo TIA			0·71
No	2888	2·4 (2·0, 3·0)	
Yes	184	2·8 (1·2, 6·9)	
No. of antiplatelets at baseline			NC
0	1997	2·4 (1·8, 3·1)	
1	976	2·4 (1·6, 3·6)	
>=2	99	NC	
Type of randomised comparator			0·0013
Aspirin/dipyridamole	1109	4·0 (2·7, 6·1)	
Clopidogrel	1430	1·8 (1·3, 2·4)	
Systolic BP			1·00
<= 140	1362	2·5 (1·8, 3·4)	
140 - 160	1115	2·5 (1·7, 3·6)	
> 160	595	2·4 (1·5, 3·9)	
Time to randomisation			0·098
<= 12	310	1·2 (0·6, 2·5)	
12 - 24	646	2·3 (1·4, 3·7)	
> 24	2116	2·8 (2·1, 3·6)	
Low dose heparin			NC
Yes	6	NC	
No	3066	2·5 (2·0, 3·0)	
Thrombolysis			0·036
Yes	339	5·4 (2·4, 12·0)	
No	2733	2·3 (1·8, 2·9)	
Gastroprotection			0·47
Yes	795	2·8 (1·8, 4·2)	
No	2277	2·3 (1·8, 3·0)	
Small vessel disease			0·60
Yes	1450	2·3 (1·7, 3·2)	
No	1265	2·6 (1·9, 3·7)	
Old vascular lesions			0·37
Yes	1584	2·2 (1·7, 3·0)	
No	1131	2·8 (2·0, 4·0)	
Ipsilateral carotid stenosis			0·25
< 50%	2422	2·5 (2·0, 3·2)	
>= 50%	395	1·7 (1·0, 2·9)	
**All Patients**	**3072**	**2·5 (2·0, 3·1)**	**< 0·0001**

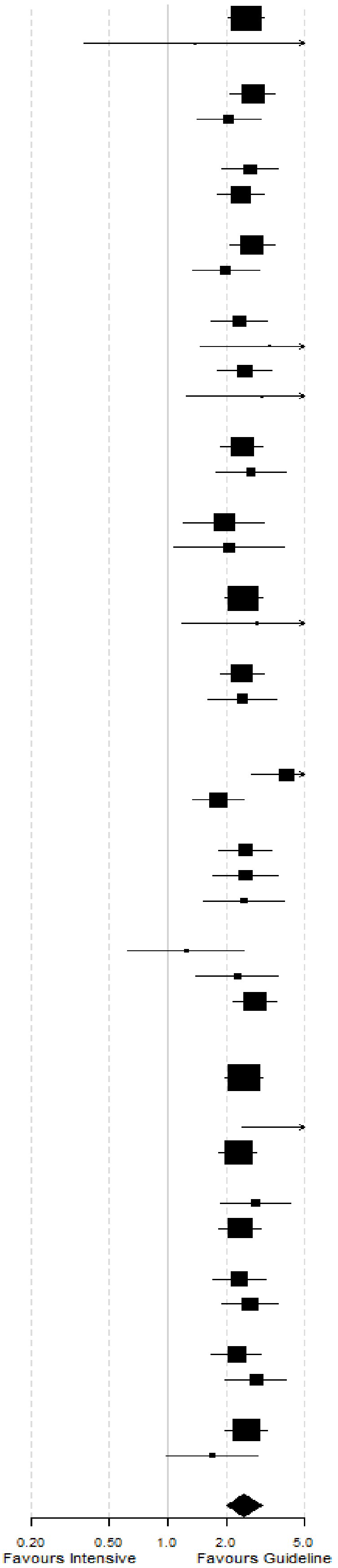

Note: 533 patients who could be randomised to either combined aspirin and dipyridamole, or clopidogrel alone, are excluded for the analysis by Type of randomised comparator.

Figure S5. Kaplan Meier plot of major bleeding.

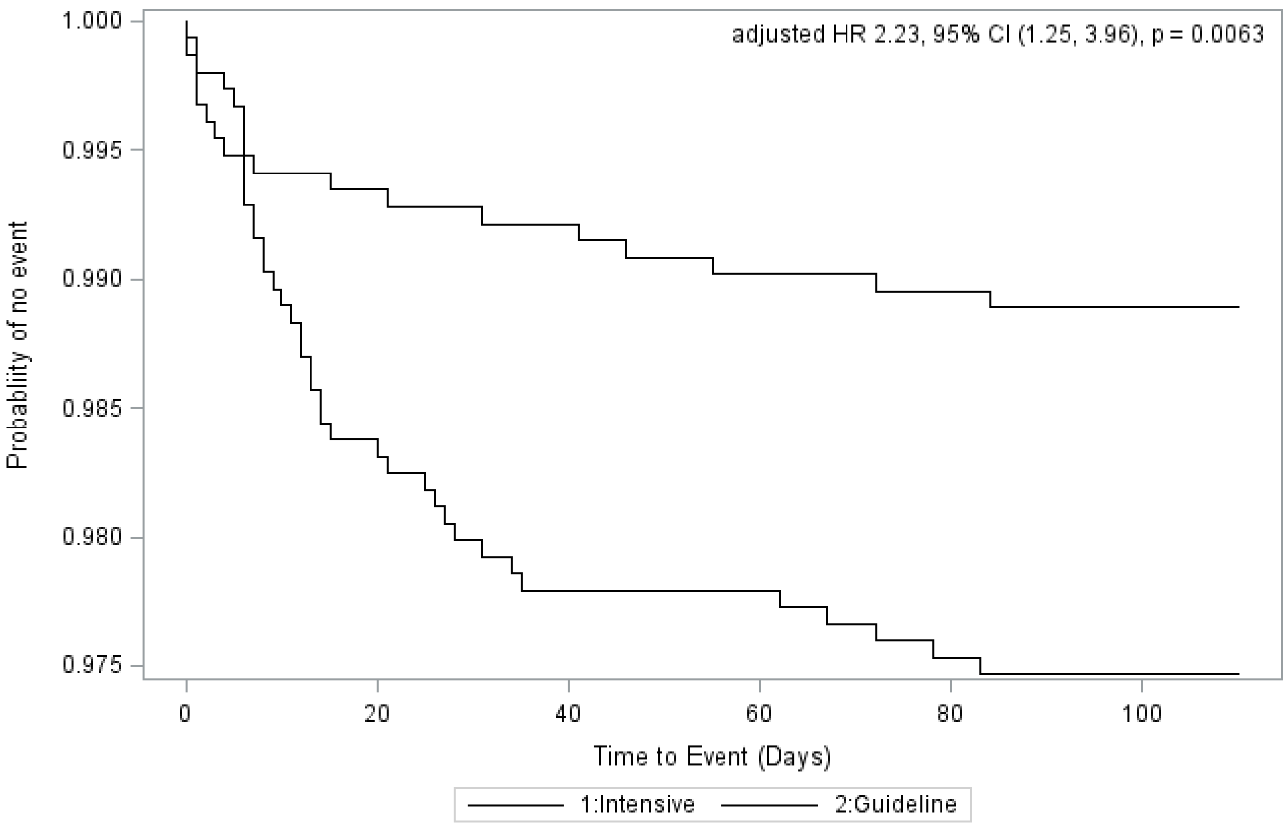

1	1540	1515	1506		1502	1501
2	1530	1520	1518	1515	1514	1513

Figure S6. Kaplan Meier of death.

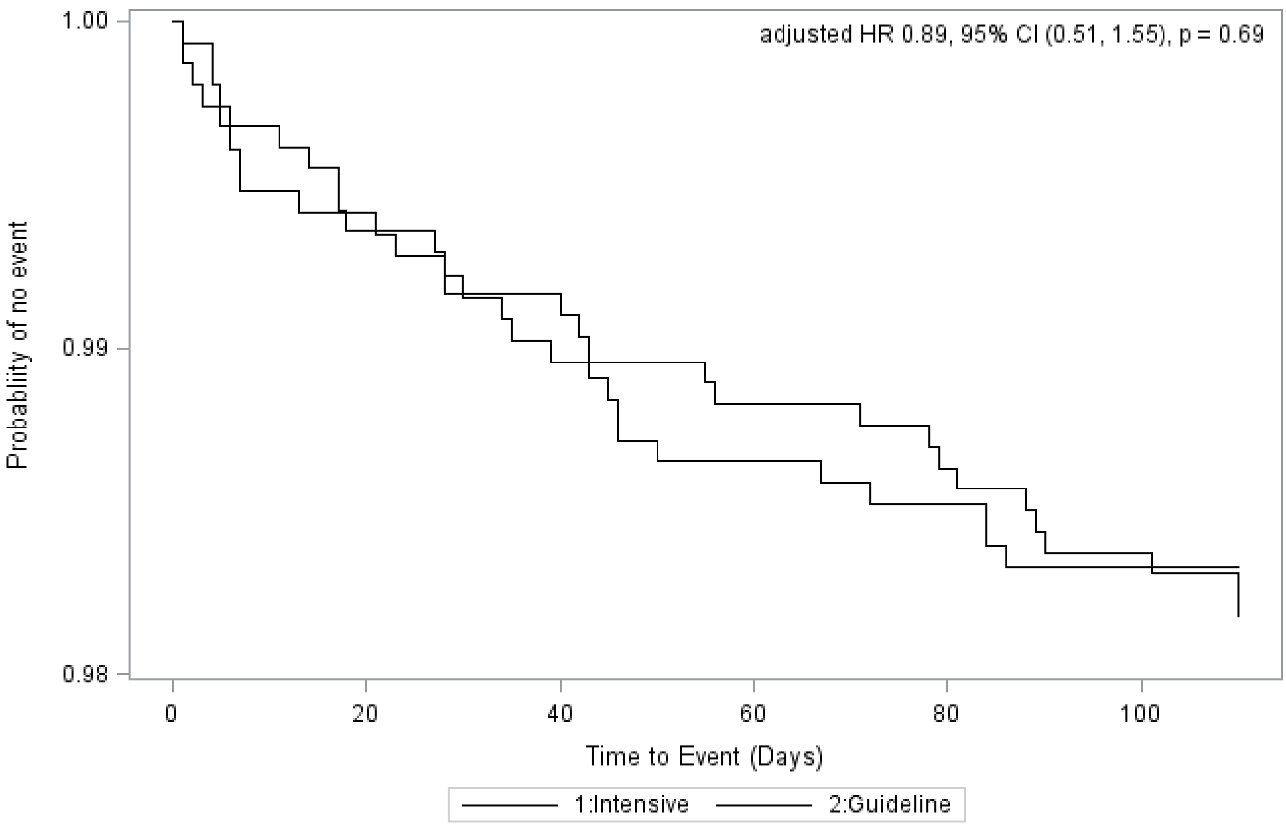

1	1556	1546	1543	1535	1533	1530
2	1535	1526	1519	1517	1514	1510

Figure S7. Distribution of serious adverse events by their severity. Severity of SAEs ordered as fatal / severe / moderate / mild / none.

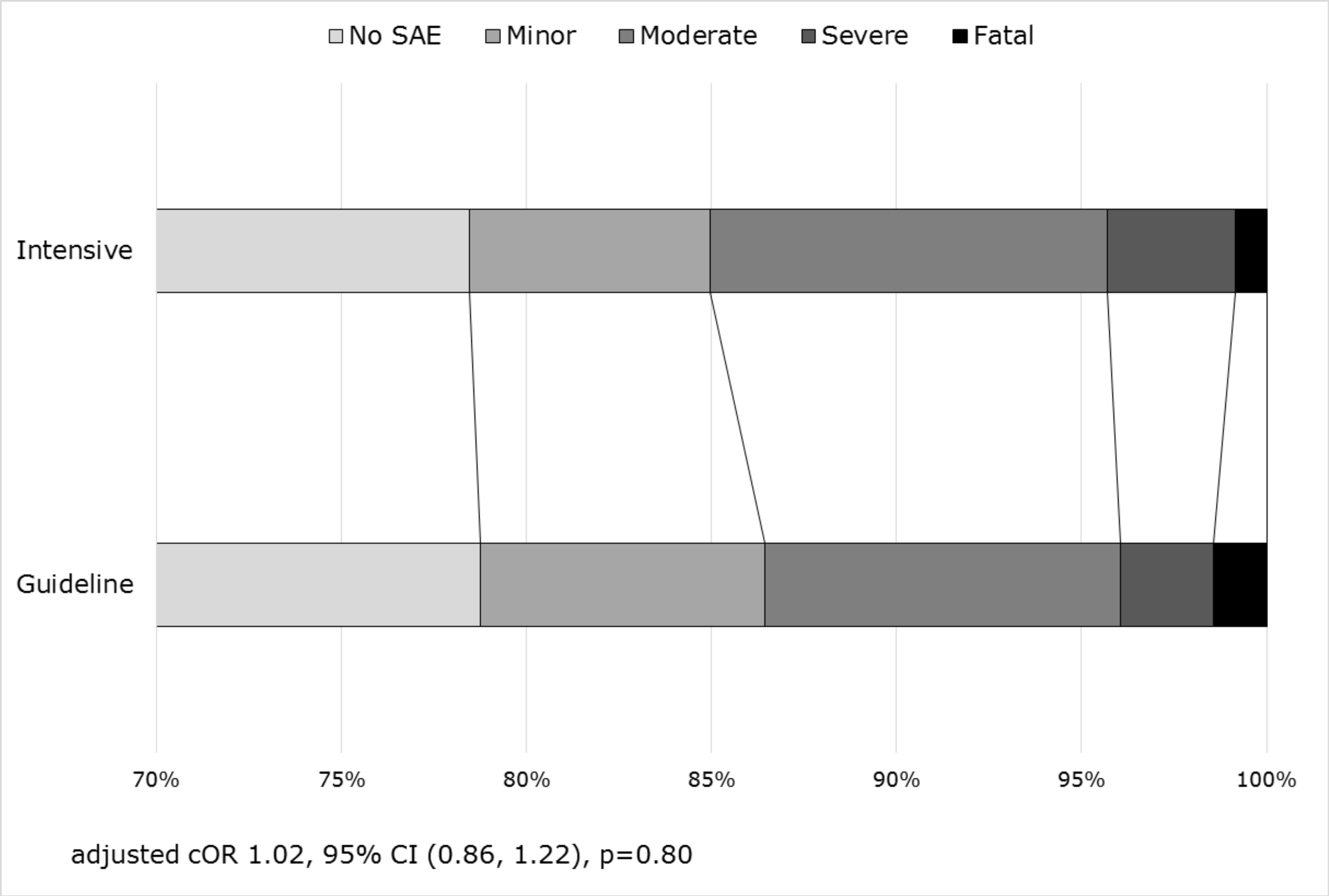

Supplement: Supplementary appendix [file mmc1.pdf]
